# Enhanced Molecular Dynamics Method to Efficiently Increase the Discrimination Capability of Computational Protein–Protein Docking

**DOI:** 10.1021/acs.jctc.1c00789

**Published:** 2021-10-15

**Authors:** Nicola Scafuri, Miguel A. Soler, Andrea Spitaleri, Walter Rocchia

**Affiliations:** CONCEPT Lab, Istituto Italiano di Tecnologia (IIT), Via E. Melen, 83, I-16152 Genova, Italy

## Abstract

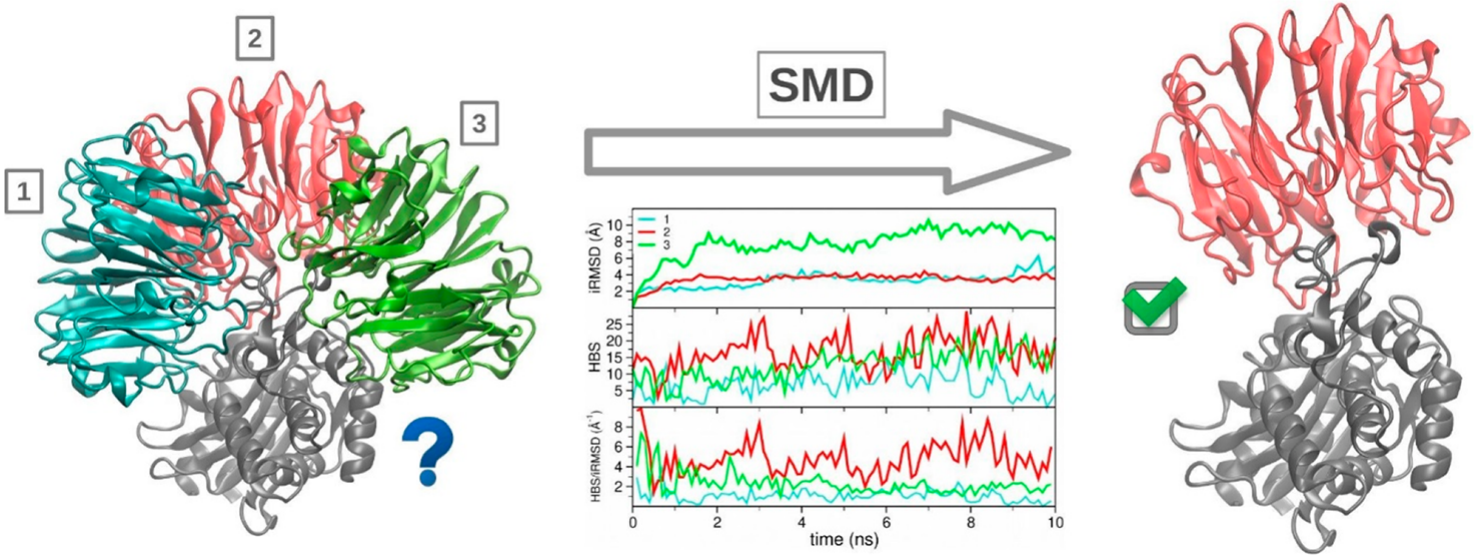

Protein–protein docking typically consists of the generation of putative binding conformations, which are subsequently ranked by fast heuristic scoring functions. The simplicity of these functions allows for computational efficiency but has severe repercussions on their discrimination capabilities. In this work, we show the effectiveness of suitable descriptors calculated along short scaled molecular dynamics runs in recognizing the nearest-native bound conformation among a set of putative structures generated by the HADDOCK tool for eight protein–protein systems.

## 1 Introduction

Protein–protein interactions (PPIs) are crucial events in biological systems needed to guarantee their correct functioning. It has been estimated^1^ that the human interactome involves between 130,000 and 600,000 PPIs.^2,3^ These interactions play a fundamental role in all the processes happening in the cell: from DNA replication to protein degradation,^4^ and perturbations in such interactions can lead to disease.^5,6^ From the biomolecular point of view, they critically involve phenomena such as recognition and binding, the deep comprehension of which represents a significant challenge.^7^ Characterizing PPIs is therefore key for a proper understanding of the mechanism underlying biological processes.^8^ Despite the fact that different techniques are used to solve protein atomic structure, such as X-ray and NMR, these can hardly be applied on a large scale; and therefore a significant number of biomolecular complexes remain beyond reach, and the structural data on protein–protein (PP) complexes in the Protein Data Bank (PDB) remain scarce because protein complexes are more difficult to crystallize than the individual proteins.^9^ Recent advances in the power of cryo-electron microscopy have allowed characterization of larger protein complexes without the need for crystallization.^10^ Moreover, many weak and/or transient PPIs that play essential roles in regulating dynamic networks in biosystems cannot be easily captured by experiments due to their unstable nature. On the computational side, there can be very diverse approaches to deal with PPI, ranging from a detailed individual molecular interaction analysis made via molecular dynamics and binding free energy calculations (MD)^11−15^ or protein–protein docking (PPD)^16^ to bioinformatics or statistical methods.^17^ PPD stands somehow in between, since it is, in principle, able to provide information at the atomistic level on an otherwise large number of interacting systems. In the same way as molecular protein–ligand docking, the PPD procedure can be basically divided into two main steps: posing and scoring. The former provides a sampling of different configurations/conformations, while the latter ranks these based on a score. The score is a very fast, and often rough, estimator of the binding strength of each pose. The score of the most stable pose is an estimate of the binding affinity of the complex.^9,18^ Despite the fact that PPD has become a reference technique because of its practical applicability, provided the atomistic structure of the binding partners is known, and affordable computational requirements, we are still far from a routine and reliable application of this technique. This is mainly because of the limited capability of sampling, especially conformational sampling, which affects the posing step, and to the limited predictivity of the scores used to perform the pose ranking.^19,20^

**Figure 1:**
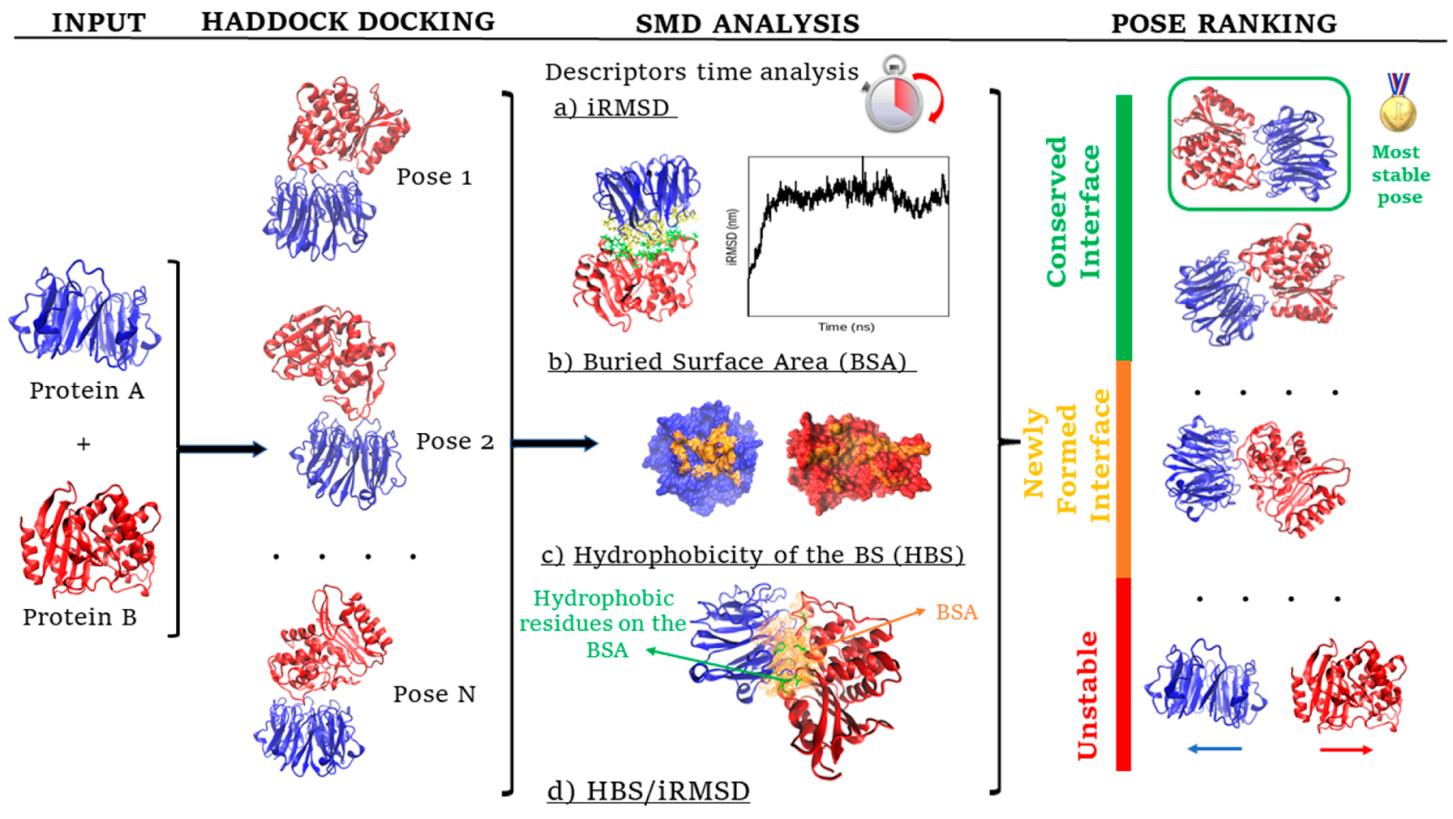
Protein–protein interacting pose ranking protocol. Candidate poses undergo a set of 3 short SMD runs where 4 descriptors are evaluated. At the end of the trajectory, the most stable poses present a conserved interface. The descriptors are iRMSD, BSA, the hydrophobicity of the BS (HBS), and the ratio of iRMSD and HBS.

In this work, we focus on the scoring step. Starting from an already published PPD benchmark done with 4 docking tools, including HADDOCK, which is one of the best performing approaches in CAPRI rounds,^21−23^ we have selected the PP systems for which near-native complexes had been generated by the posing procedure, in order to evaluate the hypothesis that an MD-based rescoring would have been able to identify them among the others. Due to the unavoidably larger computational cost inherent to the MD simulation with respect to the scoring function evaluation, a reduction of the candidate poses was obtained via an intermediate step of clustering. The detailed procedure is reported in the Materials and Methods section. We aimed to reproduce a real blind docking calculation scenario, in which the native crystallographic structure is unknown, and therefore, the scoring step is crucial in choosing the best candidate for the native structure of the complex. On purpose, we do not evaluate the ranking capability when there is no near-native pose among the set obtained filtering the results of the posing phase. This is because we do not believe that relative scoring among far-from-native poses is particularly relevant and especially that it can be evaluated by its iRMSD distance from the experimentally observed complex. It is also important to say that the present contribution is meant to be a scoring refinement and not a way to infer quantitative estimates of the free energy of binding. The idea of using scaled molecular dynamics (SMD) has already proven useful in *k*_off_-based ranking of congeneric ligands^24^ and in ranking the poses in a protein–ligand docking approach.^25^ We now assess this approach to tackle the challenging issue of ranking sampled poses in a PPD protocol. SMD is a simple, but still promising, compromise between plain MD, which provides information about the molecular determinants of biological processes, at the price of a significant computational cost, and the fast but often more inaccurate scoring functions. Here, we probe the stability of the protein–protein complexes coming from HADDOCK sampling, and we describe the performance of a set of physicochemical descriptors calculated along short SMD trajectories on PPD-derived poses (Figure 1).

Our analysis suggests that a ranking procedure based on short (5–10 ns) SMD runs can be more successful than conventional scores in identifying the near-native PPD poses at a reasonable computational cost, which is however higher than that of any scoring function evaluation. Not surprisingly, the most predictive structural descriptors are a low iRMSD, indicating the stability of the poses and the number of heavy atoms belonging to hydrophobic residues at the binding interface.

## 2 Materials and Methods

### 2.1 Choice of the PP Complexes

The systems were selected from the Updated Integrated Protein–Protein Interaction Benchmarks version 5.12. The criteria used in these benchmarks are based on the Critical Assessment of Prediction of Interactions (CAPRI) experiment, in which many computational groups test their PP complex prediction approaches.^26,27^ The benchmarks are classified based on how difficult it is to predict the correct binding pose. With respect to the previous data set composition, the most noticeable increase was for antibody–antigen complexes. More in general, it achieves a more balanced composition for most categories. For this addition, consisting of 55 new PP complexes, 400 water-refined (it1/water in HADDOCK) docking poses per system have been generated by the HADDOCK program.^28^ On that set, we performed the same protocol performed by the HADDOCK tool during its clustering step, namely a hierarchical clustering based on pairwise interface backbone root-mean-square deviation (iRMSD^B^) using the Daura algorithm^29^ with 4 Å as cutoff and discarding empty clusters.

Then, we applied the following selection criteria:

1. At least one of the cluster representatives has an iRMSD^B^ below 4 Å. This eliminates 22 out of 55 complexes.
2. In the remaining 23 systems, we selected these having at least one cluster medoid with iRMSD^B^ below 4 Å in the top 20 best scored clusters. This additional condition, introduced to limit the number of MD simulations to be performed, discards overall the 22% of the structures generated by the posing phase and leads to the final 8 protein–protein systems from the original benchmark.

The significant reduction in number is indicative of the difficulty of the 55 systems considered in this data set. The selected systems, named by the PDB code of their complex, are as follows:

1JTD:^50^ 28 kDa beta-lactamase inhibitor protein-II (BLIP-II) in complex with the TEM-1 beta-lactamase;
2YVJ:^30^ ferredoxin–ferredoxin reductase (BPHA3-BPHA4) complex;
3PC8:^31^ structure of the heterodimeric complex of XRCC1 and DNA ligase III-alpha BRCT domains;
3F1P:^32^ structure of a high affinity heterodimer of HIF2 alpha and ARNT C-terminal PAS domains;
2VXT:^33^ structure of human IL-18 complexed to murine reference antibody 125-2H Fab;
3K75:^34^ structure of reduced XRCC1 bound to DNA pol beta catalytic domain.
4H03:^35^ structure of the NAD+-Ia-actin complex;
4G6M:^36^ structure of human IL-1beta in the complex with a therapeutic antibody binding fragment of gevokizumab.

### 2.2 Scaled Molecular Dynamics (SMD)

SMD is a plain MD simulation where the potential energy and, therefore, the instantaneous forces are multiplied by a scalar, lambda < 1. We performed 3 SMD simulations on each protein–protein complex representative from the cluster analysis using the HADDOCK criteria. The setup of the SMD simulations was carried out using the BiKi Netics module of the BiKi Life Sciences suite version 1.3.5,^37^ using the API python script. We have employed the AMBER99SB-ILDN^38^ force field. Each system is built in an explicit TIP3P water box under periodic boundary conditions and, if required, neutralized by adding either Na^+^ or Cl^–^ ions. The systems followed 4 steps of equilibration going from 100 to 300 K in the NPT ensemble. Every production run of SMD is 10 ns long, using a time step of 0.002 ps with a lambda of 0.6. No restraints are applied on the systems during these runs. Long-range electrostatics interactions were calculated using the PME method.

### 2.3 Analysis of the Trajectories

Once the simulation campaign is terminated, the analysis on the stability of the PP complexes along the trajectories is carried out. In this work, the characterization of the poses was studied via the iRMSD and via some descriptors calculated at the binding interface. The analysis was performed along the entire trajectories (10 ns) considering 100 frames spaced by 100 ps. The average results have been used to build the used descriptors.

#### 2.3.1 Calculation of the PP Interfaces and the Interface Root-Mean-Square Deviation (iRMSD)

In our work, we refer to iRMSD^B^ as that calculated over the backbone of the residues at the binding interface of the interacting partners, while we refer to iRMSD as the one that also includes the side chains of these residues. In order to calculate the iRMSD along the SMD trajectories, the interface between the two binding proteins has to be defined. The interface was calculated by the PyMOl script InterfaceResidues (http://www.protein.osaka-u.ac.jp/rcsfp/supracryst/suzuki/jpxtal/Katsutani/en/interface.php). The script finds the interface residues between two proteins or binders, using the following concept: first, the Solvent Accessible Surface Area (SASA) is calculated for every residue of the binders when they are in complex and when they are isolated. A residue is defined as belonging to the binding interface if the difference of its exposed surface area between the isolated and the complex cases is greater than 1 Å^2^.

For the interface residues calculated at the initial pose, the iRMSD was calculated for each trajectory using the GROMACS package (the alignment was performed on the entire residues).^39−44^

#### 2.3.2 Calculation of the Buried Surface Area (BSA) and of the Hydrophobicity of the Buried Surface (HBS)

The calculation of the Buried Surface Area (BSA) of PP complexes was performed by means of the NanoShaper package.^45^ For our purposes, NanoShaper is called using a python script in order to perform the calculation and the analysis of the BSA of PP complexes on each frame of a SMD trajectory. For the calculation of the SASA, we have used the Solvent Excluded Surface (SES) model as implemented in NanoShaper. The probe radius was set at 1.4 Å, the grid mesh was set at 0.5 Å, and the grid profile was set at 90%.

Then, for each frame, the buried residues are classified on the basis of their nature as follows:

Hydrophobic: CYS, MET, PHE, ILE, LEU, ALA, VAL, GLY.
Neutral: SER, THR, TYR, PRO, TRP.
Hydrophilic: LYS, ASP, GLN, ASN, HIS, ARG, GLU.

This classification of the residues arises from the hydrophobicity scale proposed by Kyte and Doolittle (KD).^46^ In this scale, higher KD values indicate elevated residue hydrophobicity. We have classified as hydrophobic the residues with a KD value higher than −0.4, and we have classified as hydrophilic the residues with a KD value lower than −3.2. The rest were classified as neutral.

The HBS is therefore defined as the number of heavy atoms at the binding interface which belong to hydrophobic amino acids.

The script collects the following data about the characterization of the binding interface for each frame:

- the BSA (Å^2^), calculated as the difference between the sum of the SASAs of each solvated binder in its binding conformation and that of the PP complex;
- the number of atoms and residues on the two surfaces composing the binding interface and their characterization in terms of hydrophobicity (see above);
- the *x*, *y*, *z* coordinates of the atoms that lie at the interface;
- the IDs of the residues that lie at the interface.

In order to identify the atoms and residues that lie at the binding interface, the script uses the exposedIndices.txt file, which is where NanoShaper stores the information on the atoms that are exposed on the surface area. All the calculations are performed excluding hydrogen atoms. The script requires that the pdb files corresponding to the frames of the trajectory are extracted. This is performed using the module gmx trjconv implemented in the GROMACS package. For each trajectory (10 ns), we have extracted 100 frames spaced by 100 ps. In addition, the input files of NanoShaper are also required, in particular, *surfaceconfiguration.prm*, the file that contains all the input parameters of NanoShaper, and the .xyzr file, which contains radii and atom coordinates. It is obtained from the .pdb and amber.siz files using the pdb2xyzr script, which can be obtained from the same web site where NanoShaper can be downloaded [https://concept.iit.it/downloads]. Based on our observation, we define a further descriptor, which is the ratio between the HBS and iRMSD.

### 2.4 Validation of the SMD-Based Protocol

#### 2.4.1 Differences between Plain MD and SMD Simulations

We performed plain MD simulations on 4 systems (1JTD, 2YVJ, 3PC8, and 3F1P; 20 poses for each system, 80 simulations overall), and we compared them with the corresponding SMD simulations by analyzing the iRMSD with respect to the initial pose. As shown in Figure S1, in which a histogram of the iRMSD average values over the trajectories for both MD and SMD simulations is shown. As expected, one can clearly notice the different complex stability in the two cases: 85% of the MD trajectories have iRMSD average values concentrated in a short interval (1–3 Å). A time length of 10 ns of MD simulation is not enough to distinguish stable from metastable poses. In contrast, iRMSD average values in SMD simulations are distributed in a larger interval (1–10 Å) and lead to a significant number (more than 35%) of perturbed poses (iRMSD > 5 Å).

#### 2.4.2 Analysis of the Influence of the Lambda Parameter

We performed 3 different SMD simulations for each pose of the complexes 3K75, 4H03, and 2VXT by employing lambda values of 0.45 and 0.75, which are equidistantly located (±0.15) from 0.6. In Figure S2, we show the distribution of the iRMSD average values for all of the 162 corresponding trajectories. At lambda 0.75, more than 70% of the poses have iRMSD values of 2–3 Å, which indicates that such scaling parameter is too gentle to create the perturbation necessary to significantly probe the candidate protein–protein poses. On the other extreme, the use of lambda 0.45 triggered the detachment in more than 95% of the poses, including the near-native ones for each complex and reaching iRMSD average values above 6 Å. Using these lambda values improved no prediction.

While it is to be expected that the optimal value of lambda could depend on the size of the systems, their folding stability, and the strength of their binding, it nevertheless seems that lambda = 0.6 is a good choice and allows the correct prediction for a quite heterogeneous set of protein–protein complexes.

#### 2.4.3 Analysis of the Influence of the Time Length of SMD Simulations

Before analyzing the influence of the SMD time length, it is necessary to stress that the original aim of this work is to suggest a method which is more predictive, but still comparable in terms of computational time, with the usage of end-point scoring functions. Therefore, a compromise between computational cost and accuracy must be reached.

We extended the analysis of the behavior of the binding poses in the complex 1JTD until 30 ns. The analysis of the iRMSD allows measuring the similarity of the binding conformations within the trajectory with respect to the initial pose, while the BSA gives a direct measure of the size of the contact interface between proteins in each complex conformation. In Figures S3 and S4, we show the behavior of these descriptors over time in some representative trajectories. We can identify some common behaviors: at very short times (about 1 ns), the system is too close to its initial pose, and the perturbation is, in general, not able to provide relevant information. Consistently, iRMSD values in this time range are quite similar among different poses (see Figure S3). Similarly, BSA values at these short times indicate that the rearrangement (or the unbinding) of non-native poses has not yet occurred (see Figure S4). It appears that the most informative time window is around 5–10 ns. Here, the explored conformations are still correlated to the original pose but start showing a repertoire of different behaviors which can be used for our ranking purposes. Finally, at too long a time, the accumulation of the perturbations is degrading the information content, triggering partial unfolding and leading to very different and low-informative conformations. This can be inferred, for instance, from the increment of BSA values at times greater than 15 ns (see Figure S4). The partial unfolding events and rearrangements were confirmed by visual inspection of the structures at late stages of the trajectories (see Figure S5).

According to our analysis, the relevant information is therefore contained in the second phase, where one can observe the behavior of the system challenged by the SMD when it is still reminiscent of the starting pose. While this time window could be system dependent, based on what we observed, it seems to be located around the 5–10 ns range, which also has an affordable computational cost.

### 2.5 Ranking of the Poses

The figures employed to assess the performance of the prediction method in individuating the nearest-native binding poses are (i) the coincidence between the best ranked and the nearest-native pose (Min to Min) and (ii) the ability to position the actual nearest-native pose within one descriptor standard deviation (σ) distance from the best ranked pose and the ability to position the nearest-native pose within the first quartile. The value used for σ in this evaluation is the maximum standard deviation observed for a given descriptor throughout all the trajectories that showed some stability, that is those in which the binding interface is conserved. Based on this criterion, the σ values employed were 10 for the HADDOCK score, 0.5 Å for iRMSD, 50 Å^2^ for BSA, 1.5 atoms for HBS, and 1.6 Å^–1^ for HBS/iRMSD.

## 3 Results

### 3.1 Our Benchmark: Eight Representative PP Systems

We test our proposed pose scoring method, summarized in Figure 1, on a number of selected candidate poses (see section 2.1). The method uses three short, 10 ns, runs of SMD per pose. The scaling in the potential weakens the interactions in each simulated complex and increases its instability (see the Materials and Methods section for more details). The combination of stability and the physics of the interactions, estimated according to indicators described in section 2.3, is then used to identify the nearest-native binding pose. In 7 cases out of 8, the difference, in terms of iRMSD^B^ vs crystal, between the first and the second ranking poses (i.e., cluster representative) is greater than 2.8 Å, suggesting that there was no overlap between the nearest-native pose and the remainder of them. The diversity of the initial poses is large enough to assess the value of our procedure. In only one case, 2YVJ, this difference is very small (0.5 Å). As mentioned, for every system, we consider only the poses that are the representative structures (i.e., medoids) of the 20 best performing clusters originated from the poses in the work of Vreven et al.,^12,16^ which is based on pairwise iRMSD^B^. More details on the selection criteria can be found in the Materials and Methods section.

### 3.2 The Destabilization Induced by SMD Is Different on the Different Poses

We classified the evolution of poses during an SMD trajectory according to three different possibilities (see Figure 1): (i) the pose remains substantially stable, keeping its initial interface (conserved interface pose); (ii) the initial binding conformation is lost, and a new conformation is formed (newly formed interface pose); and (iii) the pose is unstable, and an increasing separation of the two partners is observed (unstable pose). This classification was performed by employing two descriptors: the interface root-mean-square deviation (iRMSD) with respect to the initial conformation and the buried surface area (BSA) at the interface (see the Materials and Methods section). The iRMSD is used to distinguish the conserved interface poses from the unstable ones or from those where a new interface is observed. In this way, iRMSD somehow encompasses the information carried on by iRMSD^B^ and the fraction of native contacts (FNAT, one of CAPRI’s evaluating criteria). On the basis of the investigated systems and considering the standard practice in the evaluation of PPD results, we used a 4.0 Å threshold for iRMSD to identify a conserved interface, while for values above 5.5 Å, we assumed that the initial binding conformation is lost. In the latter case, if the iRMSD stabilizes before the end of the trajectory, we say that a “new interface” is found, to keep open the, however remote, possibility that the system finds a lower energy configuration in such a short time. In the iRMSD range between 4.0 and 5.5 Å, the nature of the poses has been confirmed by visual inspection. Unstable poses show average values of BSA lower than 200 Å^2^ and high values of iRMSD (greater than 14 Å).

The data summarized in Table 1 show that in the majority of the runs the interface is conserved (from the 53% of the 4G6M system to the 88% of the 3K75), while the unstable ones are almost absent (14 unstable in 462 runs). This result confirms the ability of the HADDOCK docking protocol to generate (meta)stable poses. For all the newly formed interface poses, we have calculated the iRMSD with respect to the X-ray structure along the trajectory. In all of the cases, the X-ray-iRMSD curves showed an increasing trend (data not shown). Thus, none of the newly formed interface poses evolved toward a conformation closer to the native structure than the starting one.

**Table 1:** Stability Classification of the Different Selected Poses for the 8 Protein–Protein Systems^a^

system (PDB-ID)	no. of SMD runs	conserved interface	newly formed interface poses	unstable poses
1JTD	60	37	18	5
2YVJ	60	47	12	1
3PC8	60	50	10	0
3F1P	60	43	16	1
2VXT	42	28	11	3
3K75	60	53	6	1
4H03	60	49	11	0
4G6M	60	32	25	3

a For every initial pose, i.e., a cluster representative, 3 SMD runs have been performed and analyzed in terms of binding interface behavior, via iRMSD and BSA.

### 3.3 Identification of near-Native Poses via iRMSD and HBS

The main descriptors that have been used to characterize the interacting pair under the effects of SMD are the iRMSD and several variations of the BSA. While the iRMSD is ideal to establish the stability along the trajectory, the BSA calculation was expanded to characterize the composition of the interface itself. To do this, we extracted 6 descriptors from the BSA and assessed their value in best identifying the nearest native pose among all the stable ones. They are a) the average number of hydrophobic residues and b) the corresponding number of heavy atoms; c) the average number of hydrophilic residues and d) the corresponding number of heavy atoms; and e) the average number of neutral residues and f) the corresponding number of heavy atoms. The number of atomic contacts on the two interacting surfaces has already been shown to be a good predictor for protein–protein binding affinity.^47,48^ SMD calculations allowed us to determine that the average number of heavy atoms belonging to hydrophobic residues on the BSA, a quantity that we dubbed HBS, was the best performing indicator of the nearest-native protein–protein complex, better than considering the entire BSA as described in the work of Kastritis and co-workers.^20^ The rank of the nearest-native cluster representative poses for all 8 PP complexes based on the HADDOCK scoring function and on our three indicators calculated along the SMD trajectories is shown in Table 2. All the values of the HADDOCK scoring functions for each PP complex are reported in Table S1 in the Supporting Information. The ranking provided by the HADDOCK score, which was successful in only 1 out of the 8 examined systems, reflects the limits of scoring functions in finding the nearest-native pose among a set of candidates. Conversely, performing SMD simulations on each pose leads to a significant improvement, since all three of the best descriptors improve the scoring function’s result, as the HBS descriptor is able to map the best rank to the best pose in 4 cases out of 8. All the values of the descriptors along the SMD trajectories for each PP complex are reported in Tables S2–S5 in the Supporting Information.

**Table 2:** Ranking Comparison among HADDOCK Score, Average Buried Surface Area (BSA), Average iRMSD, Average HBS, and HBS/iRMSD Values along SMD Simulations^a^

no.	binder A	binder B	PDB	HADDOCK scoring	BSA	iRMSD	HBS	HBS/iRMSD
1	beta-lactamase inhibitor	TEM-1 β-lactamase	1JTD	4	4	10	1	1
2	BPHA3 ferredoxin	BPHA4 ferredoxin	2YVJ	2	4	2	4	3
3	XRCC1	DNA ligase III-α	3PC8	1	8	1	3	1
4	HIF2 alpha	ARNT C-terminal	3F1P	12	1	3	1	1
5	interleukin-18	antibody 125-2H Fab	2VXT	4	1	3	1	1
6	reduced XRCC1	DNA polymerase β	3K75	8	15	1	15	6
7	NAD+	Ia-actin	4H03	6	5	3	6	2
8	IL-1beta	Ab binding fragment of gevokizumab	4G6M	3	1	1	1	1

a Out of 8 systems, HADDOCK achieved success in 1, BSA in 3, iRMSD in 3, HBS in 4, and HBS/iRMSD in 5 systems.

It is interesting to note that in two cases, 1JTD and 3PC8, the prediction ranking performed by the BSA descriptor is significantly improved by the HBS-based descriptor. This confirms the importance of hydrophobicity characterization and that the burial of hydrophobic groups is more relevant than direct electrostatic interactions.^49^ In order to assess the importance of the evaluation of the descriptors along the SMD trajectory, we have also calculated the BSA and the HBS for the initial poses (Table S6 in the Supporting Information). The results clearly show that the BSA and HBS of the initial conformations are much less predictive than the same indicators averaged over the SMD trajectories. In addition to the iRMSD and BSA (Tables S2–S5 in the Supporting Information), other alternative descriptors have been tested. Their performance is summarized in Tables S7 and S8 in the Supporting Information.

**Figure 2:**
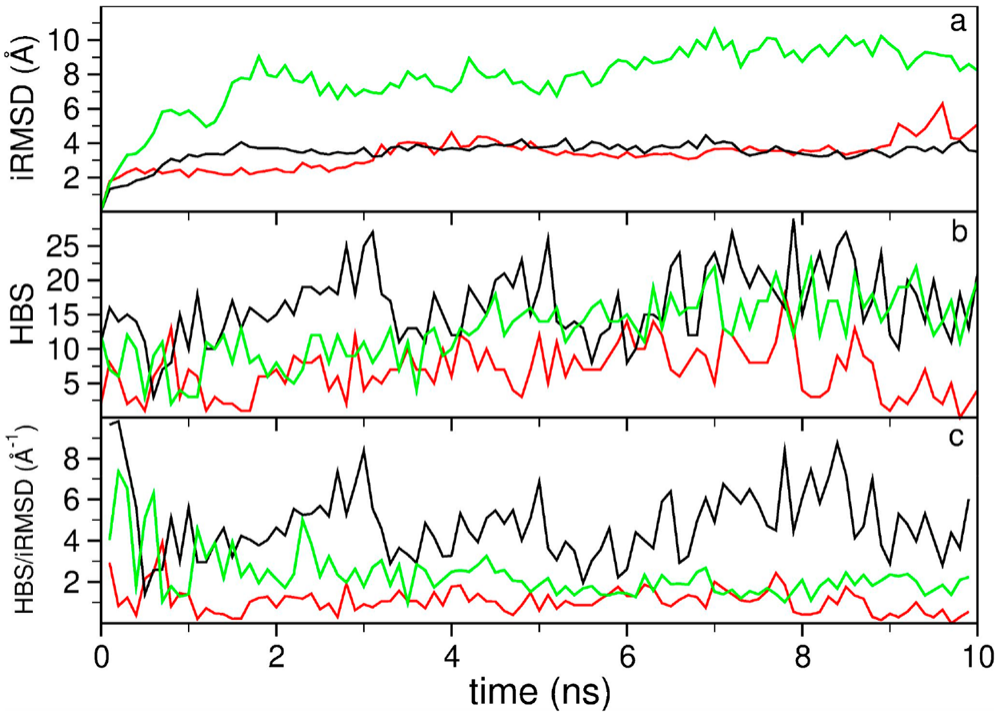
Evolution of the descriptors iRMSD (Å), HBS (number of heavy atoms), and HBS/iRMSD (Å^–1^) for the near-native pose (black curve) and two representative examples (green and red curves) of non-native poses in the complex 1JTD.

To better understand the origin of the 4 prediction failures, we more deeply evaluated the iRMSD and HBS behavior of the protein–protein complexes along the corresponding SMD trajectories. In Figure 2, we show three representative examples of the evolution of three different poses of the complex 1JTD that can depict the typical behaviors that we have recurrently found in our analysis. Near-native poses show, in general, low and steady iRMSD values, as the near-native binding conformation is more resistant to the perturbation, and high values of HBS (see the black curves in Figure 2a,b). However, other non-native poses can also show a stable behavior in some trajectories, showing iRMSD values similar to those of the near-native pose (see the red curve in the Figure 2a) at least for simulated times shorter than 10 ns. However, their HBS values are significantly lower than the ones obtained for the near-native pose (see Figure 2b). It was also observed that non-native poses can evolve during the simulation and find other (meta)stable conformations along the SMD. This situation can be clearly identified by the high iRMSD values (above 5–6 Å–see the green curve in Figure 2a). However, if the new binding conformations involve a large number of hydrophobic residues, then the HBS may achieve high values similar to those of the near-native pose (see Figure 2b). This phenomenon can be enhanced if, at the final stages of the trajectory, some local regions of the proteins unfold (see Figure S5), since they expose more previously buried hydrophobic residues. In order to have a visual description of this phenomenon, we include in the Supporting Information a video of a trajectory where this kind of rearrangement takes place.

Based on these observations, we defined a new descriptor that consists of the ratio between HBS and iRMSD (see Figure 2c). The basic idea is to evaluate the HBS, while the complex has not yet drifted too much from the initial conformation. The new descriptor is able to predict correctly the native structure of 5 out of 8 complexes (see Tables 2 and S5). In the three problematic complexes, i.e., 2YVJ, 3K75, and 4H03, their near-native structures are ranked in the third, sixth, and second position (out of 20 poses), respectively. These results significantly outperform the usage of a scoring function, albeit at a larger computational cost.

Compelled by the consideration on the computational cost, and also to explore the robustness of the approach, we repeated our analysis by employing only the first 5 ns of the SMD trajectories. We observed that halving the simulations only minimally affects the quality of the predictions. The same 5 out of 8 native complexes are still retrieved, while the near-native structures of 2YVJ, 3K75, and 4H03 systems are ranked slightly worst, at the 5th, 12th, and 10th position, respectively. These results have been included in the Tables S9–S12 to allow the user to choose the length of the SMD simulations also according to his/her computational resources.

**Figure 3:**
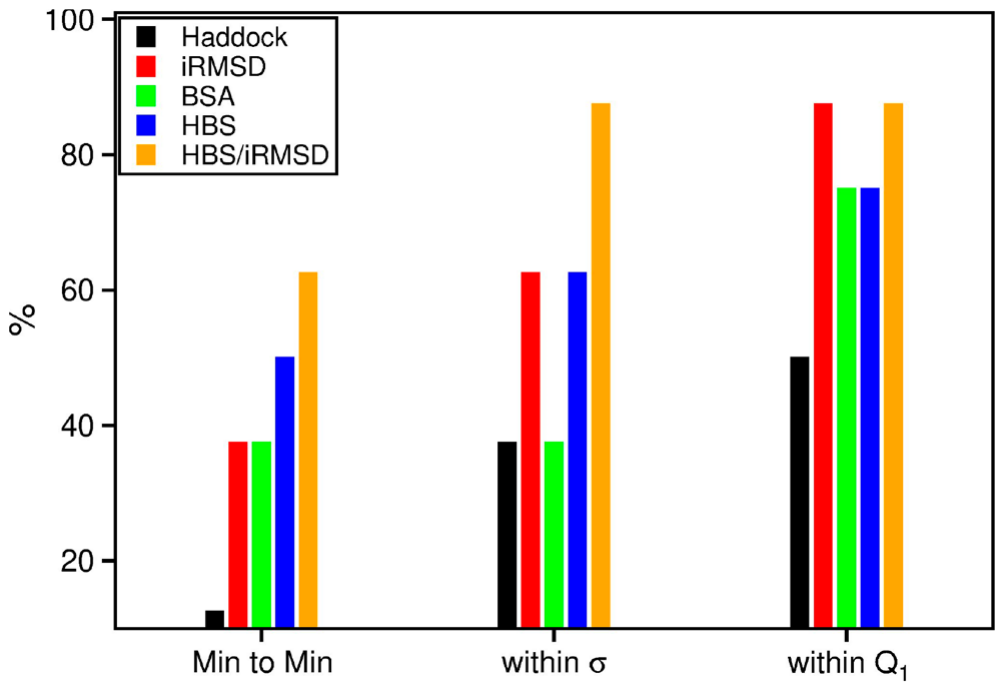
Ranking of the putative bound poses: performance of the three SMD-based descriptors and of the HADDOCK docking scoring function in identifying the nearest-native binding pose. Three figures of merit were used: the ability to map the best rank to the best configuration (Min to Min), the ability to position the actual best configuration within a standard deviation (σ) of the best ranked pose, and the ability to position the best configuration within the first quartile.

In Figure 3, the general performance of each descriptor is illustrated. We used three different figures of merit: (i) the frequency of finding the nearest-native as the best-ranked pose (min-to-min), (ii) the frequency of finding it within a window centered in the first ranked pose and having a width equal to the standard deviation of the used descriptors (see the Materials and Methods section), and (iii) the frequency of finding it within the first quartile. The HBS/iRMSD ratio appears to be the best predictor according to all three criteria and in achieving the 87.5% of success for the least strict criterion. Although the HBS descriptor performs slightly better than iRMSD and BSA in finding the nearest-native pose, the other ranking criteria seem to indicate that all three descriptors are equivalent. Despite its very good performance, HBS/iRMSD still failed to rank the nearest native pose in the first position in three systems, namely 2YVJ, 3K75, and 4H03. This is further explored in the following section.

### 3.4 The 2YVJ, 3K75, and 4H03 Cases

The three systems where our predictors fail to map the nearest-native pose to the first place in ranking correspond to the structures classified in the Protein Data Bank as 2YVJ, 3K75, and 4H03. In the 2YVJ and 3K75 systems (ferredoxin reductase BPHA4 bound to biphenyl dioxygenase ferredoxin subunit and reduced XRCC1 bound to DNA pol beta catalytic domain), no representative of the clusters of putative binding poses presents a FNAT greater than 0.2 (the FNAT of their nearest-native poses are 0.19 and 0.1, respectively) which is the limit below which CAPRI considers the protein–protein model as incorrect.^26,27^ This is a strong indication that if the compared structures are too distant from the native one, their relative ranking becomes more difficult, but, we may add, also less relevant.

**Figure 4:**
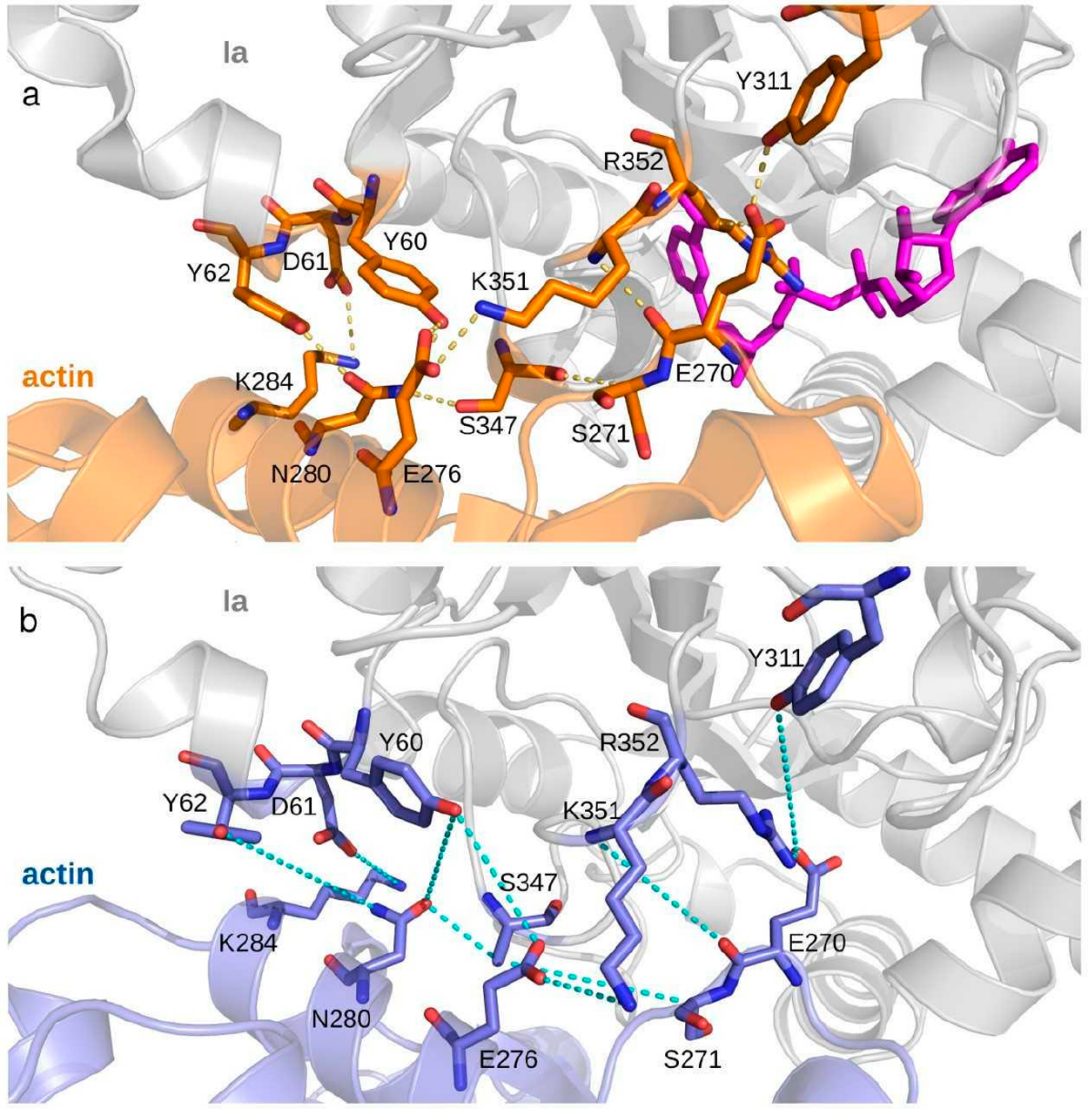
Comparison of the ionic and hydrogen bond distances between (a) the 4H03 crystal structure and (b) its nearest-native pose.

The case of the 4H03 system (NAD^+^-Ia-actin complex) is more interesting. This system has a binding interface characterized by 10 different ionic and hydrogen bonds (see Table 3). Although the number of FNAT of the best cluster representative is 0.3, we observed that only one ionic bond is conserved between that pose and the actual crystal structure of the complex (see Table 3 and Figure 4). All of the other bonds that are present in the crystal are actually missing from that pose. When the latter undergoes SMD, the weakness of the overall interaction leads to instability.

**Table 3:** Residue–Residue Contacts between Ia and Actin in the 4H03 Complex and in the Best-Ranking Pose According to the iRMSD^B^ Figure

Ia-actin native contacts	contact distance in the crystal (Å)	contact distance in the best considered pose (Å)
Y60-E276	2.6	6.1
Y60–N280	3.2	7.9
D61-K284	3.1	5.5
Y62–N280	2.6	9.9
Y311-E270	2.6	9.5
S347–S271	3.3	8.1
S347–N280	2.8	3.7
K351-E270	2.8	8.0
K351-E276	2.8	5.0
R352-E270	3.4	2.7

## 4 Discussion and Conclusions

The method presented here addresses the main hurdles faced by the *in silico* identification of protein–protein bound structures. Indeed, computational simulation of protein–protein complex formation is a daunting and, in many cases, unfeasible approach, requiring massive time and computational resources.^16^ Protein–protein docking in this respect is a fast approach, which generates putative interacting conformations and ranks them using simplified scoring functions. In the past decade, several techniques have been developed, from simulation-based to machine learning ones. However, recognizing near-native structures from a huge pool of alternatives entails a quite challenging trade-off between computational cost and accuracy. In this respect, scoring functions represent the fastest approach, but they also leave a great deal of space for improvements in accuracy. The results presented herein indicate that a suitable MD-based approach can prove useful in refining the ranking obtained using conventional scoring functions, at a reasonable computational cost (performing about 50 ns/day for an 80,000-atom and 30 ns/day for a 200,000-atom system on a dual 18-cores Intel Xeon E5-2697 architecture). The inherent computational cost made necessary a further reduction step, which we addressed by clustering the starting docking poses. In this case, the identification of the nearest-native cluster representative can be, in principle, followed by a further exploration of the other members of the cluster, to find better poses. It must be clarified that the approach proposed here is not aiming at obtaining a quantitative estimate of the binding affinity of a pose. Moreover, it is worth mentioning that some further preprocessing of the structures is still required for running the MD, requiring more effort than what is needed for evaluating a scoring function, especially in cases where some atoms or residues of the systems are missing from the resolved pdb structures, such as loops and mobile side chains. The continuous improvement of automatized software tools for homology modeling or loop reconstruction is expected to gradually reduce manual intervention in the future. This approach carries the additional advantage of being divisible into trivially parallel short simulations. Similarly to what has already been shown in the case of protein–ligand systems,^24^ this method relies on the idea that the residual stability in a perturbed dynamic simulation of two interacting proteins can be a good indicator of the “near-nativeness” of a complex. As it is well established, a key role in this respect is played by the residues participating in the binding interface region.^12,16,17^ Generally, for a protein–protein complex, a larger interface area implies that receptor and ligand can form more favorable interactions at the interface, and this is highlighted by the residual stability during the SMD simulations. In addition to the stability of the binding interface, its composition and, in particular, the average number of heavy atoms belonging to hydrophobic residues enhance the ranking of the poses generated by the PPD protocol. This is somehow in agreement with the improvement in ranking performance of scoring functions that underweight the electrostatic contribution.^37^ Overall, our approach, based on SMD and adopting the descriptor HBS/iRMSD, was able to correctly identify the nearest-native poses among a set of putative complex structures in the 62.5% of the studied systems, a significant improvement over the performance of the HADDOCK scoring function. Interestingly, we also observe that the nearest-native pose is positioned in the first quartile of the ranks in 7 out of 8 systems. The limited number of explored systems is due to the filtering criteria we applied to the initial benchmark set, which require that the previous steps of PPD provide at least one near-native pose among the representatives of the 20 best ranked clusters. This reflects our original belief, confirmed by the results of the 2YVJ and 3K75 cases, according to which this method is not suitable to ascertain whether in a set there is a near-native pose or not.

In summary, this method represents a very promising contribution to the protein–protein docking field and points to three main aspects which deserve to be considered for a better scoring of the putative binding poses, namely (i) to make use of average descriptor values along a SMD trajectory, (ii) to consider the number of heavy atoms in the buried surface which belong to hydrophobic amino acids as a necessary ingredient of a good predictor for the best pose, and (iii) to include the heavy atoms of the side chains in the calculation of the iRMSD of a PP structure. This approach paves the way for a more effective exploitation of the computational and simulative tools in a field that, as demonstrated by the CAPRI initiative, can have a strong impact on biology and medical sciences.

## Supporting Information Available

Histogram of iRMSD average values obtained from MD and SMD simulations for complexes 1JTD, 2YVJ, 3PC8, and 3F1P; distribution of iRMSD average values in complexes 3K75, 4H03, and 2VXT; evolution of iRMSD values along trajectory in 3 different poses of complex 1JTD; evolution of BSA values along trajectory in 3 different poses of complex 1JTD; binding conformations of complex 1JTD at initial and final times of trajectory; comparison of iRMSD X-ray and HADDOCK docking scoring functions in identification of nearest native pose; comparison of descriptors along SMD trajectories; comparison of descriptors on initial poses with iRMSD X-ray values; assessment of alternative binding descriptors; hydrophobic scales; and comparison of descriptors along 5 ns SMD trajectories (PDF)
Video: ExceedingRearrangement.mpg. SMD trajectory of complex ferredoxin reductase BPHA4 bound to biphenyl dioxygenase ferredoxin subunit (PDB: 2YVJ) (due to SMD, binding interface rearranges and exposes, in final part of trajectory, more hydrophobic residues (in green) than these that were present in original pose) (MP4)

## Abbreviations

PP: protein–protein
PPI: protein–protein interaction
PPD: protein–protein docking
MD: molecular dynamics
SMD: scaled molecular dynamics
iRMSD: interface root-mean-square deviation
BSA: buried surface area
HBS: hydrophobicity of the buried surface
SASA: solvent accessible surface area
FNAT: fraction of native contacts
