# Supplementary material for: Enhanced Molecular Dynamics Method to Efficiently Increase the Discrimination Capability of Computational Protein–Protein Docking
Source: J Chem Theory Comput. 2021 Oct 15;17(11):7271–80. doi: 10.1021/acs.jctc.1c00789 (PMC8582249; doi:10.1021/acs.jctc.1c00789)
Supplement: Supplementary file 1 — ct1c00789_si_001.pdf [file ct1c00789_si_001.pdf]

Enhanced molecular dynamics method to efficiently increase the discrimination capability of computational protein-protein docking

Nicola Scafuri. Miguel A. Soler. Andrea Spitaleri[†]*. Walter Rocchia**

CONCEPT Lab. Istituto Italiano di Tecnologia (IIT). Via E. Melen. 83 I-16152 Genova (Italy).

† IRCCS San Raffaele Scientific Institute, Milan, Italy

Supporting Information

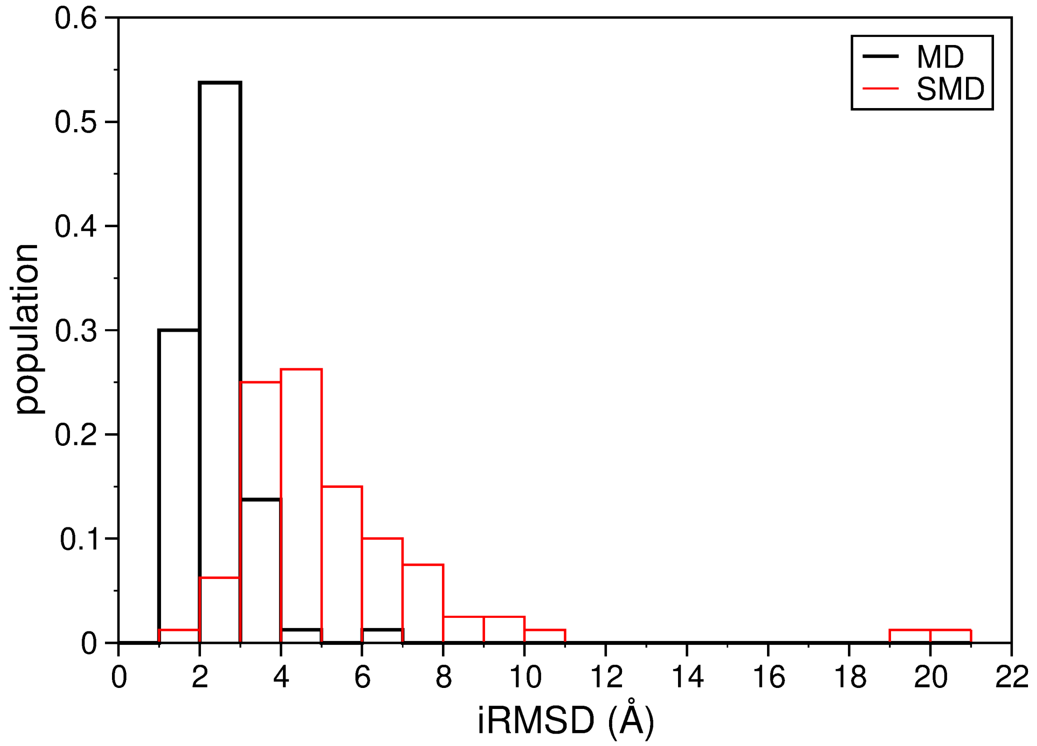

Figure S1. Histogram of the iRMSD average values obtained from MD and SMD simulations for complexes 1JTD, 2YVJ, 3PC8, and 3F1P.

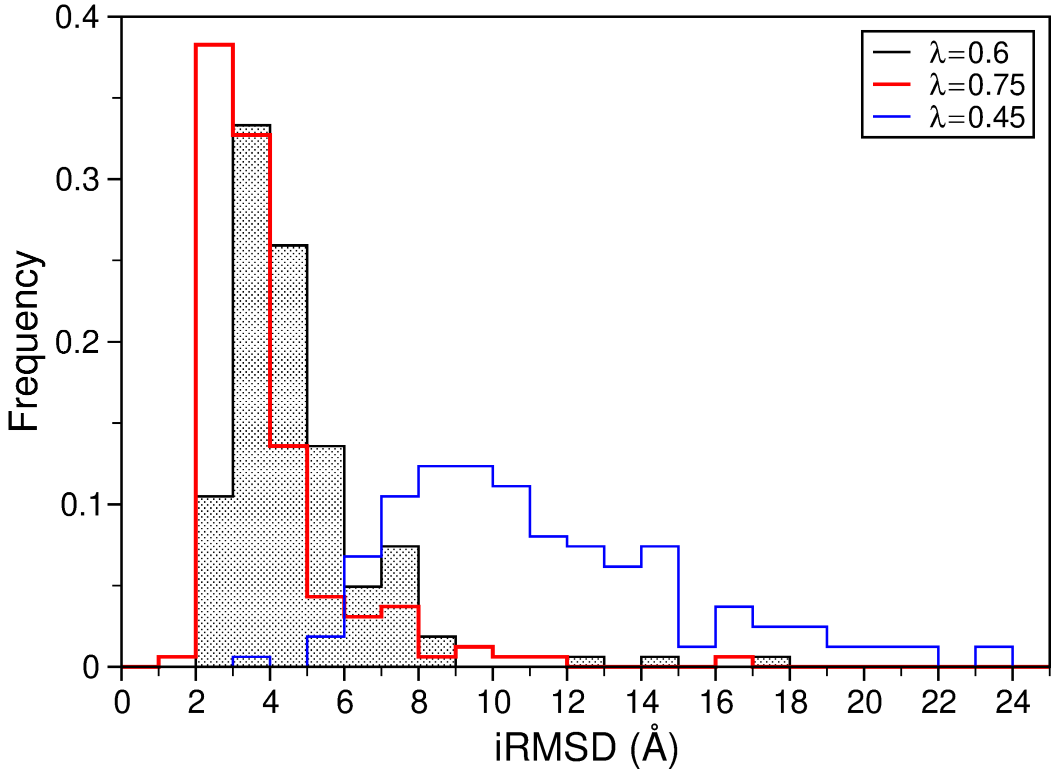

Figure S2. Distribution of the iRMSD average values in the complexes 3K75, 4H03 and 2VXT.

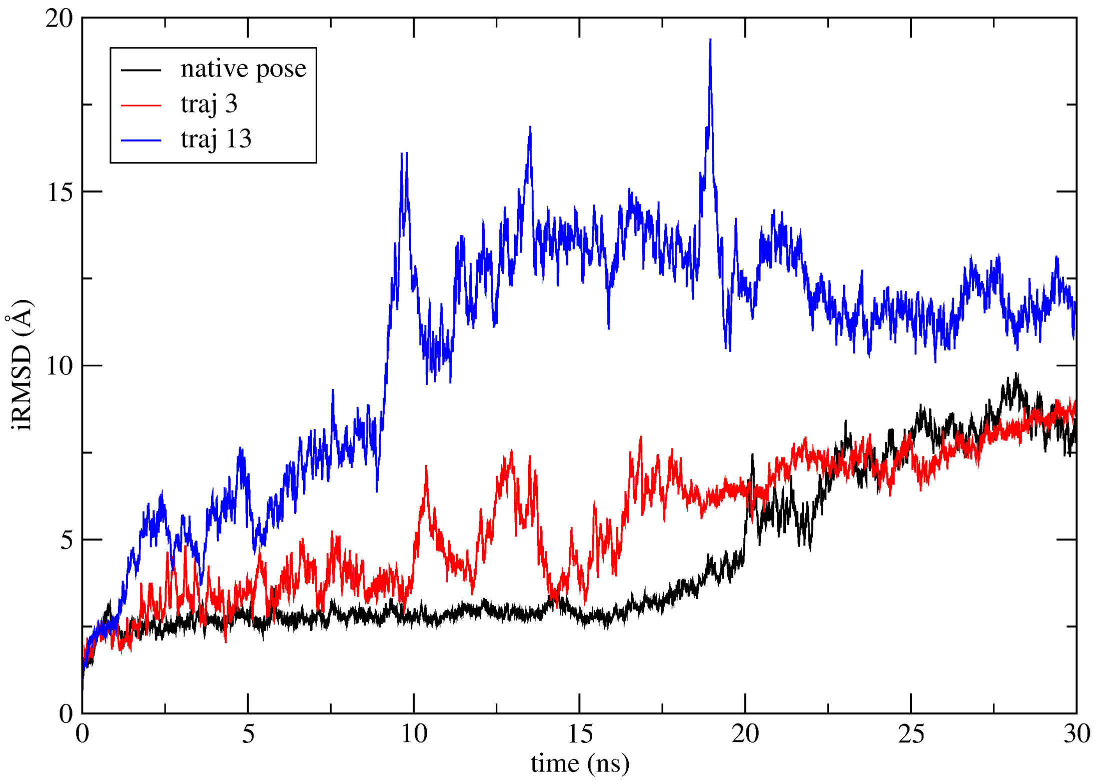

Figure S3. Evolution of iRMSD values along the trajectory in 3 different poses of the complex 1JTD.

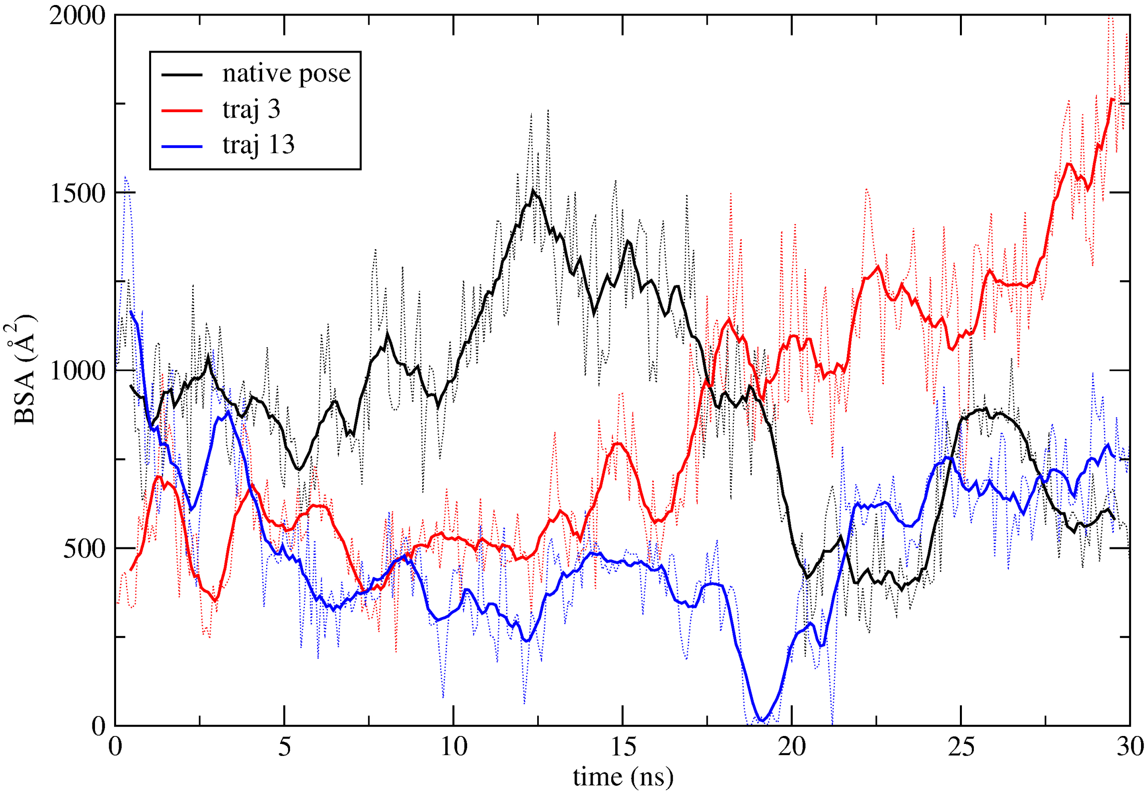

Figure S4. Evolution of BSA values along the trajectory in 3 different poses of the complex 1JTD.

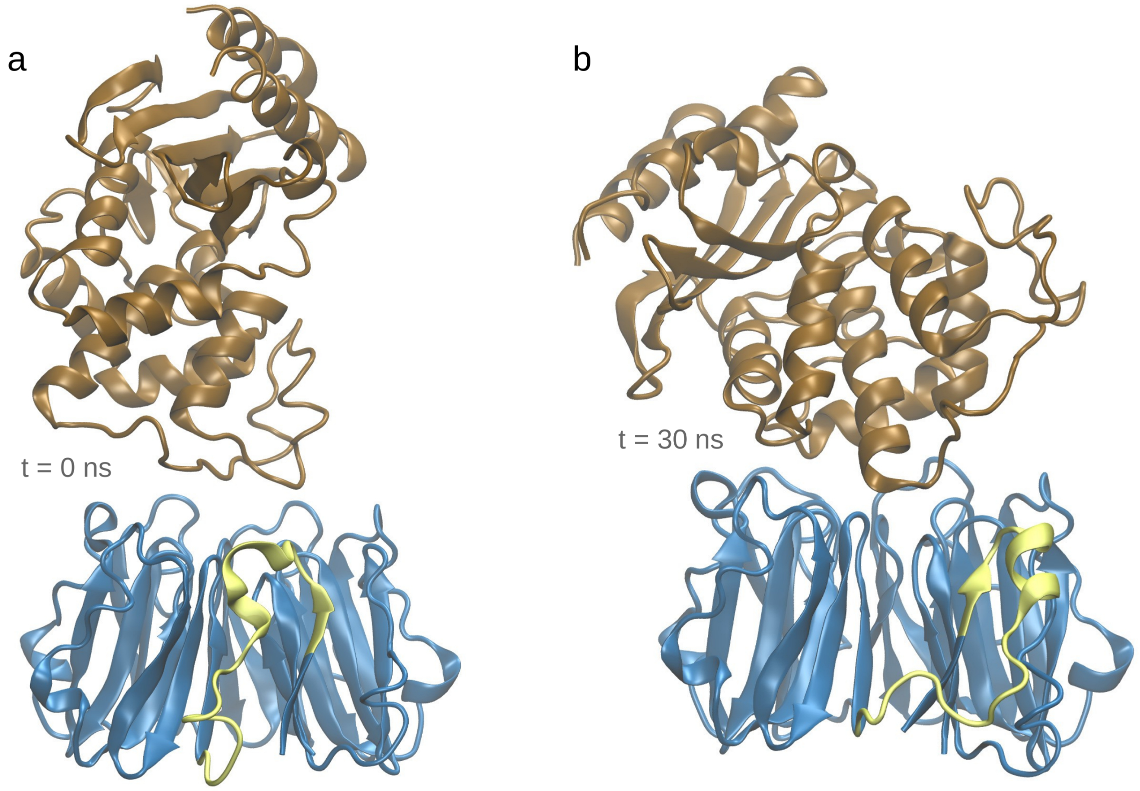

Figure S5. Binding conformations of complex 1JTD at the initial and final times of the trajectory. The protein region that partially unfolds at the final stages of the trajectory is highlighted in yellow.

Comparison of the iRMSD X-Ray and HADDOCK docking scoring functions in the identification of the nearest native pose

In Table S1 are summarized the results of the pose scoring performed with HADDOCK [1,2] and the iRMSD^B wrt. the X-ray, in accordance to the CAPRI criteria [3,4]. Here, we refer to iRMSD^B as the iRMSD calculated on the backbone atoms at the interface, while we refer to iRMSD as that including also the heavy atoms of the side chains, and that is here calculated with respect to the initial pose along the SMD trajectory. We exploited the integrated protein-protein interaction benchmarks dataset available here: https://data.sbgrid.org/dataset/131/ consisting in 55 full HADDOCK docking models data.[5] For each PP investigated system, 20 poses were taken into account, except for the system 2VXT, for which 14 poses were considered. All the poses are ordered in the table, according to their iRMSD^B, from the lowest, the nearest-native pose (highlighted in yellow) to the highest. Also, the more negative values of the scoring function, the ones that should point to the nearest-native pose, are highlighted in yellow. The analysis in Table S1 clearly shows the problems that a scoring function may have in identifying the nearest-native binding pose. Indeed, the lowest value of the scoring function corresponds to the lowest value of iRMSD^B in only 1 case out of 8 (3PC8).

***Table S1**: iRMSD^{B} and HADDOCK scoring function values for each medoid of the series of the investigated PP complexes. All the poses are ordered according to the iRMSD^{B} values, from the lowest (highlighted in yellow) to the highest. Also, the more negative values of the scoring functions are highlighted in yellow.*

PDB-ID	Pose Number	iRMSD^{B} (Å)	HADDOCK Scoring
1JTD	1	1.77	-82.34
1JTD	7	3.88	-46.23
1JTD	9	4.33	-80.26
1JTD	3	8.38	-63.36
1JTD	14	11.40	-75.92
1JTD	4	11.51	-82.08
1JTD	19	12.23	-57.65
1JTD	20	12.75	-56.48
1JTD	6	13.96	-97.82
1JTD	18	14.15	-70.46
1JTD	8	14.88	-65.01
1JTD	16	14.90	-69.66
1JTD	11	15.45	-83.04
1JTD	12	16.03	-47.46
1JTD	10	16.51	-63.09
1JTD	15	16.76	-82.00
1JTD	13	17.24	-87.52
1JTD	5	17.32	-58.36
1JTD	2	17.40	-57.94
1JTD	17	18.01	-24.90
**PDB-ID**	**Pose Number**	**iRMSD^{B} (Å)**	**HADDOCK Scoring**
2YVJ	24	3.43	-88.78
2YVJ	40	3.86	-42.72
2YVJ	21	5.35	-130.38
2YVJ	36	5.53	-71.40
2YVJ	35	7.49	-71.90
2YVJ	29	7.93	-82.54
2YVJ	30	9.31	-80.62
2YVJ	26	18.04	-62.66
2YVJ	31	18.71	7.29
2YVJ	38	18.79	-14.11
2YVJ	33	18.86	-24.67
2YVJ	37	18.99	-71.37
2YVJ	34	19.12	-28.52
2YVJ	23	19.58	2.12
2YVJ	22	19.75	-35.86
2YVJ	32	19.76	-41.48
2YVJ	28	19.85	1.01
2YVJ	39	20.09	-1.79
2YVJ	25	20.48	-88.20
2YVJ	27	21.37	-20.06
**PDB-ID**	**Pose Number**	**iRMSD^{B} (Å)**	**HADDOCK Scoring**
3PC8	41	1.02	-118.86
3PC8	52	4.84	-75.22
3PC8	55	9.70	-57.96
3PC8	49	9.98	-41.30

3PC8	45	10.30	-20.43
3PC8	56	10.61	-51.82
3PC8	50	10.81	-75.63
3PC8	51	11.42	-67.08
3PC8	43	11.79	-42.71
3PC8	47	11.93	-40.40
3PC8	59	12.06	-42.70
3PC8	60	12.93	-62.60
3PC8	42	13.05	-60.04
3PC8	44	13.82	-48.40
3PC8	46	14.05	-72.41
3PC8	57	14.29	-67.51
3PC8	53	14.40	-22.01
3PC8	48	14.45	-81.34
3PC8	54	14.46	-51.53
**PDB-ID**	**Pose Number**	**iRMSD^B (Å)**	**HADDOCK Scoring**
3F1P	72	2.95	-54.30
3F1P	69	7.89	-87.14
3F1P	74	8.64	-80.59
3F1P	67	9.01	-89.33
3F1P	61	10.01	-23.04
3F1P	70	11.13	-37.30
3F1P	65	11.29	-56.99
3F1P	80	12.06	-77.90
3F1P	64	13.28	-44.58
3F1P	77	13.39	-58.86
3F1P	75	13.69	-27.19
3F1P	76	13.75	-42.67
3F1P	68	13.99	-55.86
3F1P	71	14.02	-7.44
3F1P	79	14.08	-79.83
3F1P	66	14.60	-61.09
3F1P	62	14.83	-98.98
3F1P	78	14.88	-41.18
3F1P	73	15.23	-44.71
3F1P	63	15.83	-96.27
**PDB-ID**	**Pose Number**	**iRMSD^B (Å)**	**HADDOCK Scoring**
2VXT	10	2.51	-111.53
2VXT	12	7.11	-86.74
2VXT	6	9.17	-79.84
2VXT	14	10.30	-33.15
2VXT	7	10.42	-57.29
2VXT	3	11.20	-134.29
2VXT	1	11.48	-63.07
2VXT	13	11.73	-100.78
2VXT	11	11.75	-76.07
2VXT	2	12.93	-134.56
2VXT	4	13.13	-103.67
2VXT	8	13.15	-61.83
2VXT	9	13.53	-78.38
2VXT	5	15.15	-125.59

PDB-ID	Pose Number	iRMSD[B] (Å)	HADDOCK Scoring
3K75	25	3.38	-66.54
3K75	21	6.86	-60.98
3K75	32	7.99	-52.94
3K75	27	8.15	-41.07
3K75	30	8.48	-49.46
3K75	33	8.64	-50.84
3K75	15	8.85	-63.13
3K75	34	10.48	-78.22
3K75	28	11.94	-45.62
3K75	17	12.39	-57.20
3K75	22	12.46	-1.39
3K75	24	12.94	-91.81
3K75	20	12.97	-97.53
3K75	26	13.33	-64.62
3K75	29	14.01	-85.88
3K75	23	14.50	-25.66
3K75	31	14.77	-26.05
3K75	18	15.01	-99.93
3K75	16	16.81	-111.50
3K75	19	17.73	-99.40
**PDB-ID**	**Pose Number**	**iRMSD[B] (Å)**	**HADDOCK Scoring**
4H03	42	2.99	-106.26
4H03	54	4.80	-99.89
4H03	40	6.80	-114.12
4H03	43	8.92	-127.97
4H03	38	11.19	-91.00
4H03	52	12.01	-90.63
4H03	45	12.66	-38.90
4H03	44	14.97	-101.20
4H03	35	15.04	-96.04
4H03	53	15.38	-108.43
4H03	39	15.61	-140.82
4H03	49	16.18	-101.11
4H03	50	16.20	-76.57
4H03	48	17.45	-58.84
4H03	36	17.64	-83.82
4H03	37	18.53	-44.68
4H03	51	19.20	-117.62
4H03	47	19.26	-103.68
4H03	46	21.29	-89.23
4H03	41	21.74	-100.04
**PDB-ID**	**Pose Number**	**iRMSD[B] (Å)**	**HADDOCK Scoring**
4G6M	55	3.22	-93.98
4G6M	59	7.38	-104.53
4G6M	70	7.94	-83.33
4G6M	63	9.81	-91.15
4G6M	60	10.38	-60.69
4G6M	58	12.52	-95.96
4G6M	71	12.95	-53.97
4G6M	67	13.02	-89.10

4G6M	61	13.46	-68.55
4G6M	57	13.84	-37.82
4G6M	73	14.16	-66.68
4G6M	65	14.33	-45.78
4G6M	66	14.56	-81.73
4G6M	64	14.64	-93.56
4G6M	72	14.84	-35.48
4G6M	62	14.88	-37.66
4G6M	74	15.07	-75.73
4G6M	56	15.41	-77.01
4G6M	68	15.43	-73.61
4G6M	69	16.19	-66.44

Comparison of the descriptors along the SMD trajectories

In Tables from S2 to S5 are summarized the average values of the descriptors (iRMSD, BSA, HBS and HBS/iRMSD) over the 3 SMD trajectories for each pose. The calculation of each descriptor was carried out considering a frame each 100 ps. Every descriptor was first averaged on each of the three trajectories, then averaged of the three trajectories.

Table S2: *average values of iRMSD calculated for each pose along three different SMD trajectories (iRMSD 1st traj, iRMSD 2nd traj and iRMSD 3rd traj). and iRMSD values averaged on the three trajectories (Average iRMSD). The pose number highlighted in yellow represents the nearest native poses, having the lowest value of iRMSD^B (see Table S1). The lowest values of iRMSD that identify the most stable pose are highlighted in yellow.*

PDB-ID	Pose Number	iRMSD 1st traj (Å)	iRMSD 2nd traj (Å)	iRMSD 3rd traj (Å)	Average iRMSD (Å)
1JTD	1 (native)	3.458	6.320	6.380	5.386
1JTD	2	5.632	5.511	7.813	6.318
1JTD	3	7.253	4.658	7.830	6.580
1JTD	4	19.889	14.928	10.441	15.086
1JTD	5	5.084	6.148	3.696	4.976
1JTD	6	7.062	3.374	5.733	5.390
1JTD	7	5.174	4.897	4.659	4.910
1JTD	8	9.306	3.767	5.703	6.259
1JTD	9	6.838	16.241	3.744	8.941
1JTD	10	3.552	3.496	3.594	3.547
1JTD	11	5.584	3.933	35.737	15.084
1JTD	12	7.910	8.824	6.583	7.772
1JTD	13	4.500	3.448	4.000	3.982
1JTD	14	2.963	7.735	2.803	4.500
1JTD	15	4.084	3.836	3.889	3.936
1JTD	16	3.429	4.823	2.753	3.668
1JTD	17	7.324	5.666	6.008	6.333
1JTD	18	19.859	4.210	5.302	9.790
1JTD	19	3.475	8.163	3.197	4.945

1JTD	20	2.969	4.279	4.774	4.007
**PDB-ID**	**Pose Number**	**iRMSD 1st traj (Å)**	**iRMSD 2nd traj (Å)**	**iRMSD 3rd traj (Å)**	**Average iRMSD (Å)**
2YVJ	21	4.417	5.390	3.733	4.514
2YVJ	22	3.555	3.462	4.353	3.790
2YVJ	23	3.157	3.937	6.907	4.667
2YVJ	24 (native)	3.783	4.158	3.888	3.943
2YVJ	25	4.163	4.934	4.259	4.452
2YVJ	26	6.489	3.989	3.867	4.782
2YVJ	27	3.839	6.512	3.921	4.757
2YVJ	28	7.697	8.756	7.517	7.990
2YVJ	29	3.834	4.123	4.339	4.099
2YVJ	30	4.590	4.192	5.668	4.817
2YVJ	31	3.222	4.039	8.760	5.340
2YVJ	32	5.088	12.771	4.161	7.340
2YVJ	33	8.239	4.994	5.146	6.127
2YVJ	34	5.601	4.066	11.259	6.975
2YVJ	35	8.252	2.862	3.472	4.862
2YVJ	36	4.158	4.039	3.749	3.982
2YVJ	37	4.739	3.674	4.951	4.455
2YVJ	38	6.651	7.711	5.018	6.460
2YVJ	39	5.094	5.429	4.364	4.962
2YVJ	40	4.889	5.303	4.836	5.009
**PDB-ID**	**Pose Number**	**iRMSD 1st traj (Å)**	**iRMSD 2nd traj (Å)**	**iRMSD 3rd traj (Å)**	**Average iRMSD (Å)**
3PC8	41 (native)	2.046	2.711	2.436	2.398
3PC8	42	4.062	3.479	3.801	3.781
3PC8	43	6.939	5.325	4.941	5.735
3PC8	44	4.296	4.464	4.440	4.400
3PC8	45	4.791	4.158	4.195	4.381
3PC8	46	10.116	6.179	7.272	7.856
3PC8	47	3.325	5.518	4.356	4.400
3PC8	48	2.628	5.682	2.781	3.697
3PC8	49	7.025	4.684	5.078	5.596
3PC8	50	2.501	2.828	2.958	2.762
3PC8	51	4.746	5.041	4.916	4.901
3PC8	52	5.109	5.626	5.464	5.400
3PC8	53	3.956	4.318	4.417	4.230
3PC8	54	5.160	6.226	3.123	4.836
3PC8	55	4.213	5.415	8.791	6.140
3PC8	56	3.452	3.646	4.448	3.849
3PC8	57	2.768	2.431	2.408	2.536
3PC8	58	4.383	5.910	4.135	4.809
3PC8	59	4.651	5.160	3.092	4.301
3PC8	60	7.882	3.293	8.366	6.513
**PDB-ID**	**Pose Number**	**iRMSD 1st traj (Å)**	**iRMSD 2nd traj (Å)**	**iRMSD 3rd traj (Å)**	**Average iRMSD (Å)**
3F1P	61	6.084	3.181	10.079	6.448

3F1P	62	3.959	6.130	7.116	5.735
3F1P	63	5.257	4.824	4.941	5.008
3F1P	64	5.528	4.901	4.130	4.853
3F1P	65	3.608	4.771	4.338	4.239
3F1P	66	5.059	3.879	6.608	5.182
3F1P	67	4.353	4.672	3.761	4.262
3F1P	68	5.112	5.201	6.458	5.590
3F1P	69	4.590	7.013	4.550	5.384
3F1P	70	4.081	5.213	4.119	4.471
3F1P	71	9.724	8.748	4.892	7.788
3F1P	72 (native)	3.487	4.034	4.038	3.853
3F1P	73	4.526	5.043	4.783	4.784
3F1P	74	3.343	4.047	3.399	3.596
3F1P	75	6.205	4.496	3.612	4.771
3F1P	76	6.445	11.850	9.644	9.313
3F1P	77	4.765	4.722	4.714	4.734
3F1P	78	3.172	3.101	3.052	3.108
3F1P	79	4.125	4.298	5.590	4.671
3F1P	80	5.661	6.328	6.172	6.054
**PDB-ID**	**Pose Number**	**iRMSD 1st traj (Å)**	**iRMSD 2nd traj (Å)**	**iRMSD 3rd traj (Å)**	**Average iRMSD (Å)**
2VXT	1	4.168	3.645	3.662	3.825
2VXT	2	5.807	8.486	6.436	6.910
2VXT	3	5.344	5.105	5.302	5.250
2VXT	4	2.616	3.516	5.019	3.717
2VXT	5	3.640	6.774	4.185	4.866
2VXT	6	3.356	3.745	5.489	4.196
2VXT	7	2.534	31.129	7.711	13.791
2VXT	8	3.592	3.446	2.935	3.324
2VXT	9	3.438	3.450	2.953	3.281
2VXT	10 (native)	3.442	3.432	3.745	3.540
2VXT	11	5.830	4.198	12.113	7.380
2VXT	12	3.490	5.261	3.881	4.211
2VXT	13	7.882	5.421	7.588	6.964
2VXT	14	7.693	6.563	6.963	7.073
**PDB-ID**	**Pose Number**	**iRMSD 1st traj (Å)**	**iRMSD 2nd traj (Å)**	**iRMSD 3rd traj (Å)**	**Average iRMSD (Å)**
3K75	15	7.127	5.100	4.488	5.572
3K75	16	3.493	4.266	4.441	4.067
3K75	17	5.280	2.823	2.754	3.619
3K75	18	7.708	2.445	2.431	4.195
3K75	19	4.645	4.320	3.309	4.092
3K75	20	4.718	3.524	5.376	4.539
3K75	21	4.585	4.184	4.508	4.426
3K75	22	2.483	2.341	3.520	2.781
3K75	23	3.558	3.843	3.733	3.711
3K75	24	4.755	4.457	3.711	4.308
3K75	25 (native)	2.276	2.779	2.921	2.659
3K75	26	6.128	3.940	4.638	4.902

3K75	27	3.535	3.306	3.822	3.554
3K75	28	3.750	4.229	4.320	4.100
3K75	29	3.125	8.810	5.258	5.731
3K75	30	14.176	3.874	6.127	8.059
3K75	31	3.427	3.581	4.358	3.789
3K75	32	4.582	5.018	4.268	4.622
3K75	33	2.744	3.808	2.984	3.179
3K75	34	4.999	17.549	5.161	9.237

PDB-ID	Pose Number	iRMSD 1st traj (Å)	iRMSD 2nd traj (Å)	iRMSD 3rd traj (Å)	Average iRMSD (Å)
4H03	35	4.258	4.048	3.299	3.869
4H03	36	4.234	7.015	4.046	5.098
4H03	37	4.262	4.227	4.620	4.370
4H03	38	3.695	4.767	5.021	4.494
4H03	39	4.546	5.273	5.437	5.085
4H03	40	3.495	3.626	3.110	3.410
4H03	41	6.854	3.591	3.516	4.654
4H03	42 (native)	3.893	3.336	3.428	3.552
4H03	43	7.385	7.469	5.851	6.902
4H03	44	4.962	4.491	4.624	4.693
4H03	45	4.777	6.846	5.072	5.565
4H03	46	3.274	7.938	3.564	4.925
4H03	47	4.406	3.381	4.375	4.054
4H03	48	4.615	3.406	4.085	4.035
4H03	49	3.283	3.246	2.940	3.156
4H03	50	3.762	5.098	4.060	4.307
4H03	51	4.419	7.494	3.609	5.174
4H03	52	5.289	8.395	7.203	6.963
4H03	53	3.871	4.552	3.469	3.964
4H03	54	2.864	4.837	3.225	3.642

PDB-ID	Pose Number	iRMSD 1st traj (Å)	iRMSD 2nd traj (Å)	iRMSD 3rd traj (Å)	Average iRMSD (Å)
4G6M	55 (native)	2.157	3.888	2.483	2.842
4G6M	56	4.595	5.556	6.920	5.690
4G6M	57	8.809	6.705	6.483	7.333
4G6M	58	3.362	4.387	3.083	3.611
4G6M	59	5.629	3.726	5.374	4.910
4G6M	60	6.774	6.012	7.509	6.765
4G6M	61	9.120	5.816	8.284	7.740
4G6M	62	8.515	6.088	6.537	7.047
4G6M	63	4.405	4.653	4.412	4.490
4G6M	64	3.331	7.054	3.255	4.546
4G6M	65	7.122	5.268	3.531	5.307
4G6M	66	8.413	6.954	4.701	6.689
4G6M	67	22.951	7.270	11.801	14.007
4G6M	68	8.096	4.588	12.033	8.239
4G6M	69	5.130	3.834	4.953	4.639
4G6M	70	7.506	10.194	5.000	7.566
4G6M	71	3.717	2.461	4.798	3.659

4G6M	72	4.652	6.400	8.522	6.525
4G6M	73	3.118	3.791	3.176	3.362
4G6M	74	7.611	2.526	7.529	5.889

Table S3: *average values of BSA calculated for each pose along three different SMD trajectories (BSA 1st traj, BSA 2nd traj and BSA 3rd traj). and BSA values averaged on the three trajectories (Average BSA). The pose number highlighted in yellow represents the nearest native poses, having the lowest value of iRMSD^B (see Table S1). The highest values of BSA that identify the most stable pose are highlighted in yellow.*

PDB-ID	Pose Number	BSA 1st traj (Å^2)	BSA 2nd traj (Å^2)	BSA 3rd traj (Å^2)	Average BSA (Å^2)
1JTD	1 (native)	1077.478	807.288	521.179	801.982
1JTD	2	510.799	956.030	592.278	686.369
1JTD	3	425.834	683.549	854.036	654.473
1JTD	4	207.235	252.705	208.856	222.932
1JTD	5	756.326	687.606	783.369	742.434
1JTD	6	740.492	590.019	670.254	666.922
1JTD	7	681.605	502.982	790.387	658.325
1JTD	8	411.353	624.563	749.441	595.119
1JTD	9	596.855	190.657	920.417	569.310
1JTD	10	806.593	925.286	975.345	902.408
1JTD	11	457.907	813.834	33.713	435.151
1JTD	12	510.442	405.289	390.491	435.407
1JTD	13	585.866	1005.167	919.746	836.927
1JTD	14	1091.638	361.053	1036.722	829.804
1JTD	15	707.906	522.077	959.268	729.750
1JTD	16	631.176	596.917	719.266	649.120
1JTD	17	985.032	607.613	779.571	790.739
1JTD	18	109.784	613.449	466.476	396.570
1JTD	19	613.793	349.515	680.373	547.893
1JTD	20	609.489	685.261	652.084	648.945
**PDB-ID**	**Pose Number**	**BSA 1st traj (Å^2)**	**BSA 2nd traj (Å^2)**	**BSA 3rd traj (Å^2)**	**Average BSA (Å^2)**
2YVJ	21	786.841	739.272	1149.500	891.871
2YVJ	22	854.184	1014.187	556.240	808.204
2YVJ	23	788.885	904.827	340.926	678.213
2YVJ	24 (native)	1004.352	755.287	932.051	897.230
2YVJ	25	613.104	672.813	667.384	651.100
2YVJ	26	523.806	563.132	552.220	546.386
2YVJ	27	614.753	656.474	607.644	626.290
2YVJ	28	343.290	1050.889	434.234	609.471
2YVJ	29	949.766	1076.497	933.152	986.472
2YVJ	30	800.583	628.568	517.698	648.950
2YVJ	31	823.068	968.732	569.332	787.044
2YVJ	32	641.367	302.073	573.262	505.567

2YVJ	33	312.593	563.183	476.920	450.899
2YVJ	34	532.651	449.349	563.786	515.262
2YVJ	35	560.789	1162.891	1088.936	937.539
2YVJ	36	716.698	1043.052	1182.041	980.597
2YVJ	37	620.015	703.096	596.741	639.951
2YVJ	38	481.667	490.020	480.400	484.029
2YVJ	39	529.515	789.958	728.659	682.711
2YVJ	40	733.031	883.574	767.687	794.764
**PDB-ID**	**Pose Number**	**BSA 1st traj (Å^2)**	**BSA 2nd traj (Å^2)**	**BSA 3rd traj (Å^2)**	**Average BSA (Å^2)**
3PC8	41 (native)	628.423	645.619	706.425	660.156
3PC8	42	804.030	607.019	593.035	668.028
3PC8	43	614.634	567.237	486.622	556.164
3PC8	44	584.013	451.284	490.024	508.440
3PC8	45	631.868	748.405	584.511	654.928
3PC8	46	529.795	419.851	389.560	446.402
3PC8	47	560.265	452.038	704.774	572.359
3PC8	48	730.192	458.148	851.724	680.021
3PC8	49	600.548	741.260	741.981	694.596
3PC8	50	676.508	629.604	711.512	672.541
3PC8	51	449.874	375.821	424.080	416.592
3PC8	52	672.303	898.155	541.784	704.081
3PC8	53	664.600	1033.123	642.440	780.055
3PC8	54	349.756	460.931	594.189	468.292
3PC8	55	475.274	484.783	532.763	497.607
3PC8	56	515.566	540.965	604.681	553.738
3PC8	57	693.229	659.273	618.232	656.911
3PC8	58	824.198	566.389	890.364	760.317
3PC8	59	417.386	333.836	512.275	421.166
3PC8	60	546.847	710.848	565.311	607.669
**PDB-ID**	**Pose Number**	**BSA 1st traj (Å^2)**	**BSA 2nd traj (Å^2)**	**BSA 3rd traj (Å^2)**	**Average BSA (Å^2)**
3F1P	61	616.828	741.884	375.274	577.995
3F1P	62	800.677	478.029	683.440	654.049
3F1P	63	600.103	655.465	721.644	659.071
3F1P	64	538.957	626.281	762.636	642.625
3F1P	65	801.371	1002.644	912.685	905.567
3F1P	66	740.474	671.652	580.180	664.102
3F1P	67	1061.604	962.036	1046.245	1023.290
3F1P	68	592.549	781.866	716.075	696.830
3F1P	69	1022.533	927.875	791.879	914.096
3F1P	70	700.700	604.100	522.184	608.995
3F1P	71	426.873	441.979	609.063	492.638
3F1P	72 (native)	1287.078	1262.868	1033.582	1194.510
3F1P	73	825.840	848.194	823.384	832.473
3F1P	74	564.523	624.476	705.591	631.530
3F1P	75	933.194	979.909	915.124	942.742
3F1P	76	533.906	405.542	586.018	508.488
3F1P	77	873.725	733.018	700.905	769.216
3F1P	78	1090.133	1003.324	1037.418	1043.630
3F1P	79	788.051	829.439	630.619	749.370

3F1P	80	664.761	634.989	512.712	604.154
**PDB-ID**	**Pose Number**	**BSA 1st traj (Å^2)**	**BSA 2nd traj (Å^2)**	**BSA 3rd traj (Å^2)**	**Average BSA (Å^2)**
2VXT	1	734.039	1091.889	924.585	916.838
2VXT	2	347.622	433.944	341.856	374.474
2VXT	3	794.506	703.298	1004.006	833.936
2VXT	4	1083.356	562.371	612.752	752.826
2VXT	5	1065.335	467.070	897.770	810.058
2VXT	6	767.307	764.523	502.765	678.198
2VXT	7	33.440	35.124	1123.763	397.443
2VXT	8	727.617	1052.244	987.071	922.311
2VXT	9	934.650	1236.032	1179.957	1116.880
2VXT	10 (native)	1200.070	1246.050	979.479	1141.870
2VXT	11	703.645	780.521	232.766	572.311
2VXT	12	644.141	616.422	836.870	699.144
2VXT	13	509.314	540.567	597.723	549.201
2VXT	14	361.673	528.896	861.908	584.159
**PDB-ID**	**Pose Number**	**BSA 1st traj (Å^2)**	**BSA 2nd traj (Å^2)**	**BSA 3rd traj (Å^2)**	**Average BSA (Å^2)**
3K75	15	656.314	621.010	825.213	700.846
3K75	16	953.645	1197.788	1011.904	1054.450
3K75	17	961.885	1294.461	1084.630	1113.660
3K75	18	524.001	739.435	783.216	682.218
3K75	19	1313.039	1131.956	1201.859	1215.620
3K75	20	1283.679	1253.729	732.662	1090.020
3K75	21	944.433	1009.172	1169.898	1041.170
3K75	22	443.687	636.074	602.226	560.663
3K75	23	603.447	800.573	483.557	629.192
3K75	24	1194.633	1249.998	1192.545	1212.390
3K75	25 (native)	670.052	621.176	651.172	647.466
3K75	26	887.082	1066.152	1085.549	1012.930
3K75	27	495.890	494.986	529.141	506.672
3K75	28	951.888	1001.871	882.443	945.401
3K75	29	1310.038	866.487	784.415	986.980
3K75	30	625.713	1248.240	492.979	788.977
3K75	31	997.143	938.684	966.097	967.308
3K75	32	562.613	546.177	649.339	586.043
3K75	33	608.679	928.783	836.157	791.207
3K75	34	590.496	413.781	720.783	575.020
**PDB-ID**	**Pose Number**	**BSA 1st traj (Å^2)**	**BSA 2nd traj (Å^2)**	**BSA 3rd traj (Å^2)**	**Average BSA (Å^2)**
4H03	35	1010.916	1192.997	939.270	1047.730
4H03	36	1099.070	781.128	1469.576	1116.590
4H03	37	818.587	865.960	1157.906	947.484
4H03	38	1170.553	807.706	695.404	891.221
4H03	39	1246.695	1138.796	1430.127	1271.870
4H03	40	1134.316	857.878	836.486	942.893
4H03	41	1105.328	1175.277	898.314	1059.640
4H03	42 (native)	569.705	1409.884	1140.887	1040.160
4H03	43	412.933	782.787	501.981	565.900
4H03	44	1166.303	901.217	932.635	1000.050

4H03	45	708.753	938.095	762.681	803.177
4H03	46	969.455	453.517	729.460	717.478
4H03	47	925.669	900.144	1100.928	975.580
4H03	48	912.866	820.184	732.856	821.969
4H03	49	1011.616	713.493	885.371	870.160
4H03	50	1485.103	878.322	721.292	1028.240
4H03	51	943.164	561.435	665.776	723.458
4H03	52	827.138	601.757	467.806	632.234
4H03	53	1096.674	697.041	1121.417	971.711
4H03	54	913.766	610.989	931.684	818.813
**PDB-ID**	**Pose Number**	**BSA 1st traj (Å^2)**	**BSA 2nd traj (Å^2)**	**BSA 3rd traj (Å^2)**	**Average BSA (Å^2)**
4G6M	55 (native)	1424.402	634.161	1104.917	1054.490
4G6M	56	907.843	699.412	729.762	779.006
4G6M	57	510.773	489.930	619.496	540.066
4G6M	58	525.108	621.007	691.944	612.686
4G6M	59	565.594	645.648	462.839	558.027
4G6M	60	405.553	389.531	416.106	403.730
4G6M	61	283.124	334.363	676.042	431.176
4G6M	62	724.883	523.889	506.873	585.215
4G6M	63	566.680	378.752	479.281	474.904
4G6M	64	912.649	511.210	867.967	763.942
4G6M	65	487.955	486.196	430.756	468.302
4G6M	66	232.681	443.004	431.434	369.040
4G6M	67	82.905	328.017	314.151	241.691
4G6M	68	590.347	507.881	305.790	468.006
4G6M	69	563.052	765.854	493.915	607.607
4G6M	70	566.306	208.516	332.544	369.122
4G6M	71	518.282	444.138	570.841	511.087
4G6M	72	589.710	345.865	348.061	427.879
4G6M	73	720.857	499.847	683.549	634.751
4G6M	74	686.016	718.991	759.418	721.475

Table S4: *average values of HBS calculated for each pose along three different SMD trajectories (HBS 1st traj, HBS 2nd traj and HBS 3rd traj), and HBS values averaged on the three trajectories (Average HBS). The pose number highlighted in yellow represents the nearest native poses, having the lowest value of iRMSD*^{B} *(see Table S1). The highest values of HBS that identify the most stable pose are highlighted in yellow*

PDB-ID	Pose Number	HBS 1st traj	HBS 2nd traj	HBS 3rd traj	Average HBS
1JTD	1 (native)	16.574	14.693	7.921	13.063
1JTD	2	8.545	16.762	12.802	12.703
1JTD	3	6.307	9.228	15.812	10.449
1JTD	4	2.505	3.465	2.931	2.967
1JTD	5	5.723	5.475	7.356	6.185
1JTD	6	4.832	6.772	5.079	5.561
1JTD	7	8.842	8.000	12.228	9.690
1JTD	8	3.545	4.485	7.881	5.304
1JTD	9	14.960	2.119	9.594	8.891
1JTD	10	5.762	11.317	6.911	7.997
1JTD	11	8.119	7.624	0.198	5.314
1JTD	12	4.535	4.218	2.871	3.875
1JTD	13	4.386	10.495	10.594	8.492
1JTD	14	10.772	3.703	11.238	8.571
1JTD	15	8.277	3.337	13.792	8.469
1JTD	16	6.436	4.584	6.752	5.924
1JTD	17	6.842	5.347	5.376	5.855
1JTD	18	0.713	8.465	5.119	4.766
1JTD	19	8.158	6.396	8.574	7.710
1JTD	20	10.634	9.455	11.267	10.452
**PDB-ID**	**Pose Number**	**HBS 1st traj**	**HBS 2nd traj**	**HBS 3rd traj**	**Average HBS**
2YVJ	21	12.050	12.030	18.297	14.125
2YVJ	22	12.515	17.871	7.752	12.713
2YVJ	23	11.020	12.208	3.525	8.917
2YVJ	24 (native)	20.931	10.059	16.861	15.951
2YVJ	25	17.604	21.059	16.495	18.386
2YVJ	26	12.693	20.040	19.218	17.317
2YVJ	27	6.653	11.257	7.673	8.528
2YVJ	28	5.762	20.406	5.257	10.475
2YVJ	29	19.703	20.099	17.089	18.964
2YVJ	30	13.941	7.792	7.079	9.604
2YVJ	31	13.901	14.733	14.673	14.436
2YVJ	32	14.762	5.396	10.198	10.119
2YVJ	33	5.406	5.594	8.059	6.353
2YVJ	34	7.010	14.248	10.327	10.528
2YVJ	35	10.624	16.297	10.851	12.591
2YVJ	36	12.614	16.545	15.713	14.957
2YVJ	37	10.069	10.832	7.376	9.426
2YVJ	38	5.683	9.752	6.416	7.284
2YVJ	39	8.198	17.347	14.574	13.373
2YVJ	40	11.020	14.663	14.822	13.502
**PDB-ID**	**Pose Number**	**HBS 1st traj**	**HBS 2nd traj**	**HBS 3rd traj**	**Average HBS**
3PC8	41 (native)	16.525	13.465	15.485	15.158

3PC8	42	17.356	8.040	8.634	11.343
3PC8	43	12.178	11.772	10.525	11.492
3PC8	44	7.366	4.802	6.238	6.135
3PC8	45	8.149	10.287	7.970	8.802
3PC8	46	8.802	4.752	5.465	6.340
3PC8	47	11.812	5.475	11.901	9.729
3PC8	48	14.653	9.376	15.842	13.290
3PC8	49	12.119	19.871	18.257	16.749
3PC8	50	11.970	8.495	13.307	11.257
3PC8	51	6.089	4.812	4.663	5.188
3PC8	52	14.733	20.564	12.634	15.977
3PC8	53	8.693	21.149	9.238	13.026
3PC8	54	3.396	4.832	7.406	5.211
3PC8	55	7.713	8.139	11.515	9.122
3PC8	56	10.010	12.158	12.733	11.634
3PC8	57	14.(Å^2)970	13.297	12.248	13.505
3PC8	58	15.089	7.208	15.297	12.531
3PC8	59	2.990	3.149	5.901	4.013
3PC8	60	15.079	15.109	12.386	14.191
**PDB-ID**	**Pose Number**	**HBS 1st traj**	**HBS 2nd traj**	**HBS 3rd traj**	**Average HBS**
3F1P	61	13.208	16.980	6.257	12.149
3F1P	62	18.366	9.960	14.950	14.426
3F1P	63	13.584	13.000	14.277	13.621
3F1P	64	13.079	11.317	13.475	12.624
3F1P	65	10.139	17.653	15.931	14.574
3F1P	66	12.129	11.129	10.238	11.165
3F1P	67	33.356	25.109	31.644	30.036
3F1P	68	11.000	11.248	12.069	11.439
3F1P	69	25.752	27.386	18.079	23.739
3F1P	70	18.851	11.970	8.287	13.036
3F1P	71	7.950	12.446	12.297	10.898
3F1P	72 (native)	37.386	28.921	29.089	31.799
3F1P	73	15.683	15.188	14.505	15.125
3F1P	74	11.129	13.257	14.931	13.106
3F1P	75	14.406	17.901	13.069	15.125
3F1P	76	10.990	7.911	10.762	9.888
3F1P	77	11.495	14.723	10.030	12.083
3F1P	78	19.693	18.723	16.129	18.182
3F1P	79	18.604	16.208	19.327	18.046
3F1P	80	14.624	15.059	10.574	13.419
**PDB-ID**	**Pose Number**	**HBS 1st traj**	**HBS 2nd traj**	**HBS 3rd traj**	**Average HBS**
2VXT	1	6.198	8.921	5.634	6.917
2VXT	2	4.525	3.842	4.228	4.198
2VXT	3	8.238	7.455	8.792	8.162
2VXT	4	14.822	4.574	4.109	7.835
2VXT	5	16.653	1.822	14.267	10.914
2VXT	6	8.733	8.594	3.970	7.099
2VXT	7	0.089	0.050	18.574	6.238
2VXT	8	8.802	11.723	12.950	11.158
2VXT	9	10.970	13.020	12.059	12.017
2VXT	10 (native)	19.812	20.960	17.297	19.356

2VXT	11	5.604	4.277	2.921	4.267
2VXT	12	4.505	4.752	8.782	6.013
2VXT	13	3.950	4.752	8.337	5.680
2VXT	14	6.911	4.911	11.248	7.690
**PDB-ID**	**Pose Number**	**HBS 1st traj**	**HBS 2nd traj**	**HBS 3rd traj**	**Average HBS**
3K75	15	8.941	17.416	11.366	12.574
3K75	16	17.030	20.960	15.762	17.918
3K75	17	8.663	19.178	22.079	16.640
3K75	18	13.406	12.950	11.505	12.621
3K75	19	32.554	20.208	14.584	22.449
3K75	20	11.188	13.752	18.158	14.366
3K75	21	4.277	8.366	9.149	7.264
3K75	22	7.139	10.168	7.099	8.135
3K75	23	19.465	17.267	16.713	17.815
3K75	24	10.297	8.535	9.713	9.515
3K75	25 (native)	10.921	15.624	15.228	13.924
3K75	26	7.426	7.129	7.644	7.399
3K75	27	16.426	13.990	12.901	14.439
3K75	28	20.297	10.743	8.228	13.089
3K75	29	9.406	18.267	5.683	11.119
3K75	30	13.713	12.624	15.752	14.030
3K75	31	8.535	8.782	7.030	8.116
3K75	32	7.861	9.822	9.079	8.921
3K75	33	9.515	2.634	11.970	8.040
3K75	34	4.809	17.156	5.041	9.002
**PDB-ID**	**Pose Number**	**HBS 1st traj**	**HBS 2nd traj**	**HBS 3rd traj**	**Average HBS**
4H03	35	17.525	21.010	12.347	16.960
4H03	36	23.337	11.752	24.772	19.954
4H03	37	14.040	14.099	23.406	17.182
4H03	38	20.436	13.792	11.703	15.310
4H03	39	18.772	15.713	20.158	18.215
4H03	40	23.554	15.267	18.356	19.059
4H03	41	20.713	23.723	15.931	20.122
4H03	42 (native)	10.267	22.436	23.238	18.647
4H03	43	6.911	9.812	5.584	7.436
4H03	44	26.149	17.109	15.386	19.548
4H03	45	13.901	13.822	13.376	13.700
4H03	46	17.515	7.000	17.851	14.122
4H03	47	22.020	20.634	20.584	21.079
4H03	48	19.347	18.228	13.723	17.099
4H03	49	18.693	10.772	14.832	14.766
4H03	50	30.337	9.683	11.931	17.317
4H03	51	18.366	7.762	12.871	13.000
4H03	52	13.574	13.545	11.228	12.782
4H03	53	20.238	11.158	15.059	15.485
4H03	54	13.604	7.842	13.238	11.561
**PDB-ID**	**Pose Number**	**HBS 1st traj**	**HBS 2nd traj**	**HBS 3rd traj**	**Average HBS**
4G6M	55 (native)	19.683	8.198	15.109	14.330
4G6M	56	6.644	6.158	6.287	6.363
4G6M	57	3.960	4.040	8.485	5.495
4G6M	58	3.950	7.050	7.198	6.066

4G6M	59	5.327	6.050	5.812	5.729
4G6M	60	6.079	2.931	6.594	5.201
4G6M	61	2.337	0.931	5.861	3.043
4G6M	62	11.149	6.307	7.109	8.188
4G6M	63	5.673	4.287	4.089	4.683
4G6M	64	6.941	6.505	9.208	7.551
4G6M	65	3.822	4.436	3.465	3.908
4G6M	66	1.931	4.317	5.535	3.927
4G6M	67	0.693	3.822	4.277	2.931
4G6M	68	7.000	4.693	2.812	4.835
4G6M	69	8.208	7.020	5.257	6.828
4G6M	70	5.376	1.673	1.931	2.993
4G6M	71	6.594	3.317	5.347	5.086
4G6M	72	6.020	2.881	4.208	4.370
4G6M	73	12.000	6.406	10.673	9.693
4G6M	74	7.089	9.941	6.149	7.726

Table S5: *average values of HBS/iRMSD calculated for each pose along three different SMD trajectories (HBS/iRMSD 1st traj, HBS/iRMSD 2nd traj and HBS/iRMSD 3rd traj), and HBS/iRMSD values averaged on the three trajectories (Average HBS/iRMSD). The pose number highlighted in yellow represents the nearest native poses, having the lowest value of iRMSD[B] (see Table S1). The highest values of HBS/iRMSD that identify the most stable pose are highlighted in yellow*

PDB-ID	Pose Number	HBS/iRMSD 1st traj (Å^{-1})	HBS/iRMSD 2nd traj (Å^{-1})	HBS/iRMSD 3rd traj (Å^{-1})	Average HBS/iRMSD (Å^{-1})
1JTD	1 (native)	4.794	2.325	1.241	2.787
1JTD	2	1.517	3.042	1.639	2.066
1JTD	3	0.870	1.981	2.019	1.623
1JTD	4	0.126	0.232	0.281	0.213
1JTD	5	1.126	0.891	1.991	1.336
1JTD	6	0.684	2.007	0.886	1.192
1JTD	7	1.709	1.634	2.624	1.989
1JTD	8	0.381	1.191	1.382	0.984
1JTD	9	2.188	0.130	2.562	1.627
1JTD	10	1.622	3.237	1.923	2.261
1JTD	11	1.454	1.939	0.006	1.133
1JTD	12	0.573	0.478	0.436	0.496
1JTD	13	0.975	3.044	2.649	2.222
1JTD	14	3.636	0.479	4.009	2.708
1JTD	15	2.027	0.870	3.547	2.148
1JTD	16	1.877	0.951	2.453	1.760
1JTD	17	0.934	0.944	0.895	0.924
1JTD	18	0.036	2.011	0.966	1.004
1JTD	19	2.348	0.784	2.682	1.938
1JTD	20	3.582	2.210	2.360	2.717
**PDB-ID**	**Pose Number**	**HBS/iRMSD 1st traj (Å^{-1})**	**HBS/iRMSD 2nd traj (Å^{-1})**	**HBS/iRMSD 3rd traj (Å^{-1})**	**Average HBS/iRMSD (Å^{-1})**
2YVJ	21	2.728	2.232	4.902	3.287
2YVJ	22	3.520	5.162	1.781	3.488
2YVJ	23	3.491	3.101	0.510	2.367
2YVJ	24 (native)	5.532	2.419	4.337	4.096
2YVJ	25	4.229	4.269	3.873	4.123
2YVJ	26	1.956	5.024	4.970	3.983
2YVJ	27	1.733	1.729	1.957	1.806
2YVJ	28	0.749	2.331	0.699	1.260
2YVJ	29	5.138	4.875	3.938	4.651
2YVJ	30	3.037	1.859	1.249	2.048
2YVJ	31	4.315	3.648	1.675	3.213
2YVJ	32	2.901	0.423	2.451	1.925
2YVJ	33	0.656	1.120	1.566	1.114
2YVJ	34	1.252	3.504	0.917	1.891
2YVJ	35	1.287	5.694	3.126	3.369
2YVJ	36	3.033	4.097	4.191	3.774
2YVJ	37	2.125	2.948	1.490	2.188
2YVJ	38	0.855	1.265	1.279	1.133
2YVJ	39	1.609	3.195	3.340	2.715
2YVJ	40	2.254	2.765	3.065	2.695

PDB-ID	Pose Number	HBS/iRMSD 1st traj (Å^{-1})	HBS/iRMSD 2nd traj (Å^{-1})	HBS/iRMSD 3rd traj (Å^{-1})	Average HBS/ iRMSD (Å^{-1})
3PC8	41 (native)	8.078	4.966	6.356	6.467
3PC8	42	4.273	2.311	2.271	2.952
3PC8	43	1.755	2.211	2.130	2.032
3PC8	44	1.715	1.076	1.405	1.398
3PC8	45	1.701	2.474	1.900	2.025
3PC8	46	0.870	0.769	0.752	0.797
3PC8	47	3.553	0.992	2.732	2.426
3PC8	48	5.575	1.650	5.697	4.307
3PC8	49	1.725	4.243	3.595	3.188
3PC8	50	4.785	3.004	4.498	4.096
3PC8	51	1.283	0.955	0.949	1.062
3PC8	52	2.883	3.655	2.312	2.950
3PC8	53	2.198	4.898	2.091	3.062
3PC8	54	0.658	0.776	2.372	1.269
3PC8	55	1.831	1.503	1.310	1.548
3PC8	56	2.900	3.335	2.863	3.032
3PC8	57	5.408	5.470	5.087	5.322
3PC8	58	3.443	1.220	3.699	2.787
3PC8	59	0.643	0.610	1.908	1.054
3PC8	60	1.913	4.588	1.481	2.661
**PDB-ID**	**Pose Number**	**HBS/iRMSD 1st traj (Å^{-1})**	**HBS/iRMSD 2nd traj (Å^{-1})**	**HBS/iRMSD 3rd traj (Å^{-1})**	**Average HBS/iRMSD (Å^{-1})**
3F1P	61	2.171	5.338	0.621	2.710
3F1P	62	4.640	1.625	2.101	2.788
3F1P	63	2.584	2.695	2.889	2.723
3F1P	64	2.366	2.309	3.263	2.646
3F1P	65	2.810	3.700	3.673	3.394
3F1P	66	2.397	2.869	1.549	2.272
3F1P	67	7.663	5.374	8.413	7.150
3F1P	68	2.152	2.163	1.869	2.061
3F1P	69	5.610	3.905	3.973	4.496
3F1P	70	4.620	2.296	2.012	2.976
3F1P	71	0.818	1.423	2.514	1.585
3F1P	72 (native)	10.723	7.170	7.204	8.366
3F1P	73	3.465	3.012	3.033	3.170
3F1P	74	3.328	3.276	4.393	3.666
3F1P	75	2.322	3.982	3.619	3.307
3F1P	76	1.705	0.668	1.116	1.163
3F1P	77	2.412	3.118	2.128	2.553
3F1P	78	6.209	6.037	5.284	5.843
3F1P	79	4.510	3.771	3.457	3.913
3F1P	80	2.583	2.380	1.713	2.225
**PDB-ID**	**Pose Number**	**HBS/iRMSD 1st traj (Å^{-1})**	**HBS/iRMSD 2nd traj (Å^{-1})**	**HBS/iRMSD 3rd traj (Å^{-1})**	**Average HBS/iRMSD (Å^{-1})**
2VXT	1	1.487	2.447	1.539	1.824
2VXT	2	0.779	0.453	0.657	0.630
2VXT	3	1.542	1.460	1.658	1.553

2VXT	4	5.667	1.301	0.819	2.595
2VXT	5	4.575	0.269	3.409	2.751
2VXT	6	2.602	2.295	0.723	1.874
2VXT	7	0.035	0.002	2.409	0.815
2VXT	8	2.450	3.402	4.413	3.422
2VXT	9	3.190	3.774	4.083	3.683
2VXT	10 (native)	5.756	6.107	4.619	5.494
2VXT	11	0.961	1.019	0.241	0.740
2VXT	12	1.291	0.903	2.263	1.486
2VXT	13	0.501	0.877	1.099	0.825
2VXT	14	0.898	0.748	1.615	1.087
**PDB-ID**	**Pose Number**	**HBS/iRMSD 1st traj (Å^{-1})**	**HBS/iRMSD 2nd traj (Å^{-1})**	**HBS/iRMSD 3rd traj (Å^{-1})**	**Average HBS/iRMSD (Å^{-1})**
3K75	15	1.678	3.262	2.969	2.636
3K75	16	2.559	4.082	2.560	3.067
3K75	17	3.225	7.425	5.724	5.458
3K75	18	1.124	7.842	9.083	6.016
3K75	19	2.886	2.998	3.476	3.120
3K75	20	6.901	5.735	2.713	5.116
3K75	21	2.440	3.287	4.028	3.252
3K75	22	1.723	3.574	2.599	2.632
3K75	23	2.006	2.646	1.902	2.185
3K75	24	4.093	3.874	4.504	4.157
3K75	25 (native)	4.524	3.072	3.325	3.640
3K75	26	1.782	3.965	3.283	3.010
3K75	27	2.100	2.156	2.000	2.086
3K75	28	4.380	3.308	2.986	3.558
3K75	29	6.496	1.219	1.565	3.093
3K75	30	0.664	4.715	0.928	2.102
3K75	31	4.001	3.525	3.615	3.714
3K75	32	1.863	1.750	1.647	1.753
3K75	33	2.865	2.579	3.043	2.829
3K75	34	1.903	0.150	2.319	1.458
**PDB-ID**	**Pose Number**	**HBS/iRMSD 1st traj (Å^{-1})**	**HBS/iRMSD 2nd traj (Å^{-1})**	**HBS/iRMSD 3rd traj (Å^{-1})**	**Average HBS/iRMSD (Å^{-1})**
4H03	35	4.116	5.190	3.742	4.349
4H03	36	5.512	1.675	6.123	4.437
4H03	37	3.294	3.335	5.066	3.898
4H03	38	5.531	2.893	2.331	3.585
4H03	39	4.130	2.980	3.708	3.606
4H03	40	6.739	4.211	5.903	5.617
4H03	41	3.022	6.605	4.531	4.719
4H03	42 (native)	2.637	6.726	6.780	5.381
4H03	43	0.936	1.314	0.954	1.068
4H03	44	5.270	3.809	3.327	4.135
4H03	45	2.910	2.019	2.637	2.522
4H03	46	5.350	0.882	5.009	3.747
4H03	47	4.998	6.103	4.705	5.269
4H03	48	4.192	5.352	3.359	4.301

4H03	49	5.695	3.319	5.045	4.686
4H03	50	8.064	1.899	2.939	4.301
4H03	51	4.156	1.036	3.566	2.919
4H03	52	2.567	1.613	1.559	1.913
4H03	53	5.228	2.451	4.341	4.007
4H03	54	4.749	1.621	4.104	3.492

**PDB-ID**	**Pose Number**	**HBS/iRMSD 1st traj (Å^{-1})**	**HBS/iRMSD 2nd traj (Å^{-1})**	**HBS/iRMSD 3rd traj (Å^{-1})**	**Average HBS/iRMSD (Å^{-1})**
4G6M	55 (native)	9.126	2.109	6.085	5.773
4G6M	56	1.446	1.108	0.909	1.154
4G6M	57	0.450	0.602	1.309	0.787
4G6M	58	1.175	1.607	2.335	1.706
4G6M	59	0.946	1.624	1.081	1.217
4G6M	60	0.897	0.487	0.878	0.754
4G6M	61	0.256	0.160	0.708	0.375
4G6M	62	1.309	1.036	1.088	1.144
4G6M	63	1.288	0.921	0.927	1.045
4G6M	64	2.084	0.922	2.829	1.945
4G6M	65	0.537	0.842	0.981	0.787
4G6M	66	0.229	0.621	1.177	0.676
4G6M	67	0.030	0.526	0.362	0.306
4G6M	68	0.865	1.023	0.234	0.707
4G6M	69	1.600	1.831	1.062	1.497
4G6M	70	0.716	0.164	0.386	0.422
4G6M	71	1.774	1.348	1.114	1.412
4G6M	72	1.294	0.450	0.494	0.746
4G6M	73	3.848	1.690	3.361	2.966
4G6M	74	0.931	3.936	0.817	1.895

Comparison of the descriptors on the initial poses with the iRMSD X-ray values

In Table S6 the values of BSA and HBS calculated for the initial poses of each PP complex are summarized. The poses are ordered according to the iRMSD^B, in ascending order, so that they are ordered from the nearest-native one. All the descriptors that individuate the most stable pose, highest HBS and highest value of BSA, are highlighted in yellow. The results clearly show that the HBS and BSA calculated on the initial poses and not on the SMD trajectories are inadequate for ranking the nearest native pose in 7 cases on 8.

Table S6: *values of BSA and HBS calculated for the initial poses of each PP complex. For each PP complex, the poses are ordered on the base of the iRMSD^B, in ascending order. All the descriptors that identify the most stable pose, highest HBS and highest value of BSA, are highlighted in yellow*

PDB-ID	Pose Number	iRMSD^B (Å)	BSA (Å^2)	HBS
1JTD	1	1.77	830.48	11
1JTD	7	3.88	559.41	6
1JTD	9	4.33	966.48	18
1JTD	3	8.38	353.92	2
1JTD	14	11.40	691.83	7
1JTD	4	11.51	414.81	1
1JTD	19	12.26	479.11	3
1JTD	20	12.75	435.70	3
1JTD	6	13.96	489.47	2
1JTD	18	14.15	354.24	2
1JTD	8	14.87	634.03	4
1JTD	16	14.90	473.88	4
1JTD	11	15.45	486.16	0
1JTD	12	16.03	428.14	10
1JTD	10	16.51	820.23	1
1JTD	15	16.76	792.83	13
1JTD	13	17.24	957.36	7
1JTD	5	17.32	391.82	0
1JTD	2	17.40	576.32	12
1JTD	17	18.01	341.89	2
**PDB-ID**	**Pose Number**	**iRMSD^B (Å)**	**BSA (Å^2)**	**HBS**
2YVJ	24	3.43	802.71	10
2YVJ	40	3.86	1015.54	14
2YVJ	21	5.35	1309.70	14
2YVJ	36	5.53	1106.00	15
2YVJ	35	7.49	493.40	8
2YVJ	29	7.93	740.30	10
2YVJ	30	9.31	772.18	6
2YVJ	26	18.04	544.27	25
2YVJ	31	18.71	480.44	7
2YVJ	38	18.79	604.35	4
2YVJ	33	18.86	338.88	0
2YVJ	37	18.99	757.79	6
2YVJ	34	19.12	358.86	7
2YVJ	23	19.58	524.20	5
2YVJ	22	19.75	783.82	11
2YVJ	32	19.76	548.89	12

2YVJ	28	19.85	409.73	5
2YVJ	39	20.09	541.45	9
2YVJ	25	20.48	914.54	27
2YVJ	27	21.37	333.18	6
**PDB-ID**	**Pose Number**	**iRMSD^B (Å)**	**BSA (Å^2)**	**HBS**
3PC8	41	1.02	640.57	19
3PC8	52	4.84	1075.58	14
3PC8	55	9.70	309.75	5
3PC8	49	9.98	230.81	3
3PC8	45	10.30	144.10	1
3PC8	56	10.61	358.90	4
3PC8	50	10.81	579.70	11
3PC8	51	11.42	456.36	12
3PC8	43	11.79	698.35	27
3PC8	47	11.93	331.69	5
3PC8	59	12.06	395.46	0
3PC8	60	12.93	703.23	18
3PC8	42	13.05	484.41	3
3PC8	44	13.82	537.85	2
3PC8	46	14.05	414.27	3
3PC8	57	14.29	384.47	12
3PC8	53	14.40	366.99	4
3PC8	58	14.44	504.96	5
3PC8	48	14.45	677.77	16
3PC8	54	14.46	527.84	8
**PDB-ID**	**Pose Number**	**iRMSD^B (Å)**	**BSA (Å^2)**	**HBS**
3F1P	72	2.95	979.31	19
3F1P	69	7.89	889.04	28
3F1P	74	8.64	730.67	15
3F1P	67	9.01	593.70	15
3F1P	61	10.01	472.73	10
3F1P	70	11.13	515.09	6
3F1P	65	11.29	729.78	10
3F1P	80	12.06	1058.98	24
3F1P	64	13.28	819.46	17
3F1P	77	13.39	580.28	5
3F1P	75	13.69	745.38	11
3F1P	76	13.75	238.19	0
3F1P	68	13.99	458.73	7
3F1P	71	14.02	478.21	11
3F1P	79	14.08	670.63	9
3F1P	66	14.60	710.00	13
3F1P	62	14.83	369.57	4
3F1P	78	14.88	692.69	15
3F1P	73	15.23	384.28	5
3F1P	63	15.83	474.86	4
**PDB-ID**	**Pose Number**	**iRMSD^B (Å)**	**BSA (Å^2)**	**HBS**
2VXT	10	2.51	1123.03	13

2VXT	12	7.11	599.37	3
2VXT	6	9.17	406.54	6
2VXT	14	10.30	624.60	1
2VXT	7	10.42	425.53	3
2VXT	3	11.20	560.61	12
2VXT	1	11.48	1425.82	10
2VXT	13	11.73	809.72	13
2VXT	11	11.75	771.80	12
2VXT	2	12.93	939.07	16
2VXT	4	13.13	1062.63	14
2VXT	8	13.15	710.45	8
2VXT	9	13.53	1178.80	13
2VXT	5	15.15	582.66	8
**PDB-ID**	**Pose Number**	**iRMSD[B] (Å)**	**BSA (Å^2)**	**HBS**
3K75	25	3.38	458.57	8
3K75	21	6.86	787.83	12
3K75	32	7.99	595.54	12
3K75	27	8.15	174.11	0
3K75	30	8.48	292.26	2
3K75	33	8.64	472.24	5
3K75	15	8.85	933.19	20
3K75	34	10.48	267.13	5
3K75	28	11.94	477.93	8
3K75	17	12.39	968.38	16
3K75	22	12.46	419.78	2
3K75	24	12.94	906.27	27
3K75	20	12.97	956.83	23
3K75	26	13.33	1055.63	17
3K75	29	14.01	994.11	15
3K75	23	14.50	571.78	8
3K75	31	14.77	426.05	7
3K75	18	15.01	760.47	19
3K75	16	16.81	863.01	7
3K75	19	17.73	1015.68	13
**PDB-ID**	**Pose Number**	**iRMSD[B] (Å)ray (Å)**	**BSA (Å^2)**	**HBS**
4H03	42	2.99	690.90	12
4H03	54	4.80	490.00	5
4H03	40	6.80	826.10	21
4H03	43	8.92	623.50	13
4H03	38	11.19	1024.30	9
4H03	52	12.01	778.00	18
4H03	45	12.66	451.80	2
4H03	44	14.97	417.50	7
4H03	35	15.04	587.50	3
4H03	53	15.38	874.90	8
4H03	39	15.61	1150.90	22
4H03	49	16.18	907.40	9
4H03	50	16.20	613.30	7
4H03	48	17.45	406.50	14

4H03	36	17.64	656.30	19
4H03	37	18.53	219.90	1
4H03	51	19.20	315.60	5
4H03	47	19.26	746.80	14
4H03	46	21.29	686.30	18
4H03	41	21.74	854.70	11
**PDB-ID**	**Pose Number**	**iRMSD^B (Å)**	**BSA (Å^2)**	**HBS**
4G6M	55	3.22	1105.34	18
4G6M	59	7.38	831.37	14
4G6M	70	7.94	188.74	1
4G6M	63	9.81	632.34	8
4G6M	60	10.38	424.22	3
4G6M	58	12.52	397.59	6
4G6M	71	12.95	498.62	5
4G6M	67	13.02	662.91	8
4G6M	61	13.46	577.37	5
4G6M	57	13.84	398.63	3
4G6M	73	14.16	631.04	7
4G6M	65	14.33	431.58	4
4G6M	66	14.56	523.46	3
4G6M	64	14.64	805.34	13
4G6M	72	14.84	468.44	3
4G6M	62	14.88	279.23	4
4G6M	74	15.07	793.45	11
4G6M	56	15.41	201.89	2
4G6M	68	15.43	526.16	2
4G6M	69	16.19	587.11	4

Assessment of alternative binding descriptors

In addition to the iRMSD along a trajectory and the total BSA, which are widely used descriptors for the stability of a binding configuration, we tested several variations, seeking the best performing ones. In the following, we refer to the "binding interface" as the set of residues of both proteins in a PP complex that have some atoms at a distance below a given threshold. More details are provided in the Materials and Methods section.

- Binding interface contacts

This descriptor counts the number of heavy atom pairs lying at the opposite sides of the binding interface at a distance below a given threshold. This descriptor presents some overlap with the concept of the BSA. We monitor this widely used descriptor along the SMD trajectories using two different thresholds, 3 Å and 6 Å. In Table S7, we report the average values calculated along the SMD trajectories for all the systems. The contacts at 3 Å are able to correctly rank the nearest native pose for 50 % of the systems, while the 6 Å threshold led to a 37.5% success rate.

- Interface Hydrophobicity

This descriptor is calculated by scoring the hydrophobicity of all the residues participating in the binding interface. The underlying rationale is to take into account hydrophobicity, a major determinant of protein-protein binding. This quantity was considered both statically, on the initial pose. and dynamically, averaging it along the SMD trajectory. Several hydrophobicity scales have been considered: KD (Kyte and Doolittle.

1982). WW (Wimley and White. 1996). HH (Hessa et al.. 2005). MF (Moon and Fleming. 2011) and TT (Zhao and London. 2006). The results are summarized in Table S8.

- Hydrophobic residues on the BSA

Along with the HBS, we have also calculated the number of hydrophobic residues on the BSA. The performance of this reduced version of HBS is slightly worse, correctly ranking the nearest native pose in 5 PP systems out of 8 (62.5% of the cases). The average values of the hydrophobic residues on the BSA along the trajectories for the representative poses are reported in the Table S7.

Table S7*: average values of the number of contacts at the binding interface (within 3 Å and 6 Å) and the number of hydrophobic residues on the BSA, calculated for all the poses of the PP complexes along the SMD trajectories. For each PP complex, the poses are ordered on the base of the iRMSD^{B}, in ascending order. All the descriptors that identify the most stable pose, highest number of contacts and highest number of hydrophobic residues on the BSA. are highlighted in yellow.*

PDB-ID	Pose Number	iRMSD^{B} (Å)	Interface Contacts (< 3 Å)	Interface Contacts (< 6 Å)	Hydrophobic residues on the BSA
1JTD	1	1.77	103.56	826.93	11.00
1JTD	7	3.88	65.39	506.37	6.27
1JTD	9	4.33	41.48	309.72	8.65
1JTD	3	8.38	29.65	235.19	4.46
1JTD	14	11.40	86.79	770.88	6.38
1JTD	4	11.51	5.09	31.12	1.79
1JTD	19	12.26	51.17	422.19	4.76
1JTD	20	12.75	71.91	471.47	6.50
1JTD	6	13.96	69.78	575.17	2.89
1JTD	18	14.15	10.41	83.30	0.57
1JTD	8	14.87	26.45	238.85	2.48
1JTD	16	14.90	73.03	580.36	4.32
1JTD	11	15.45	53.42	513.28	5.56
1JTD	12	16.03	35.26	313.79	3.03
1JTD	10	16.51	103.06	756.54	4.42
1JTD	15	16.76	81.05	619.50	4.89
1JTD	13	17.24	72.01	567.37	2.95
1JTD	5	17.32	74.33	566.05	4.19
1JTD	2	17.40	45.25	385.04	5.41
1JTD	17	18.01	87.73	633.23	4.96
PDB-ID	Pose Number	iRMSD^{B} (Å)	Interface Contacts (< 3 Å)	Interface Contacts (< 6 Å)	Hydrophobic residues on the BSA
2YVJ	24	3.43	123.89	859.26	9.63
2YVJ	40	3.86	61.37	486.17	6.07
2YVJ	21	5.35	99.46	809.95	6.39
2YVJ	36	5.53	81.46	637.12	5.77
2YVJ	35	7.49	45.12	351.46	5.98
2YVJ	29	7.93	110.88	823.64	9.59
2YVJ	30	9.31	100.35	687.03	6.83
2YVJ	26	18.04	41.89	327.57	6.57
2YVJ	31	18.71	90.82	511.91	9.56
2YVJ	38	18.79	43.90	322.49	3.88
2YVJ	33	18.86	25.38	121.33	3.28

2YVJ	37	18.99	59.90	528.47	6.23
2YVJ	34	19.12	25.26	195.19	4.78
2YVJ	23	19.58	90.11	721.43	6.69
2YVJ	22	19.75	82.98	542.92	8.68
2YVJ	32	19.76	49.87	389.36	8.27
2YVJ	28	19.85	22.41	158.54	3.91
2YVJ	39	20.09	53.32	392.80	5.91
2YVJ	25	20.48	83.21	624.97	8.90
2YVJ	27	21.37	40.48	320.37	4.41
**PDB-ID**	**Pose Number**	**iRMSD[B] (Å)**	**Interface Contacts (< 3 Å)**	**Interface Contacts (< 6 Å)**	**Hydrophobic residues on the BSA**
3PC8	41	1.02	106.05	672.17	7.55
3PC8	52	4.84	98.80	672.77	8.44
3PC8	55	9.70	63.53	412.34	4.38
3PC8	49	9.98	73.59	491.15	6.73
3PC8	45	10.30	52.26	362.53	5.23
3PC8	56	10.61	68.25	389.47	5.62
3PC8	50	10.81	109.11	692.58	5.92
3PC8	51	11.42	62.37	447.48	4.18
3PC8	43	11.79	47.19	252.31	6.68
3PC8	47	11.93	67.70	480.33	7.03
3PC8	59	12.06	34.98	291.28	2.14
3PC8	60	12.93	49.82	382.73	6.99
3PC8	42	13.05	75.60	542.17	9.31
3PC8	44	13.82	73.46	581.77	4.25
3PC8	46	14.05	20.16	129.14	4.26
3PC8	57	14.29	87.59	662.64	7.52
3PC8	53	14.40	62.19	340.19	5.18
3PC8	58	14.44	102.88	769.91	8.01
3PC8	48	14.45	100.03	735.55	9.07
3PC8	54	14.46	57.14	378.76	1.00
**PDB-ID**	**Pose Number**	**iRMSD[B] (Å)**	**Interface Contacts (< 3 Å)**	**Interface Contacts (< 6 Å)**	**Hydrophobic residues on the BSA**
3F1P	72	2.95	142.79	812.99	14.95
3F1P	69	7.89	113.13	748.97	11.56
3F1P	74	8.64	79.79	604.19	5.48
3F1P	67	9.01	115.99	843.92	13.38
3F1P	61	10.01	60.85	413.54	7.01
3F1P	70	11.13	74.35	546.69	8.42
3F1P	65	11.29	76.13	539.11	5.79
3F1P	80	12.06	75.29	568.60	7.31
3F1P	64	13.28	54.43	426.50	6.89
3F1P	77	13.39	93.41	664.12	7.52
3F1P	75	13.69	82.67	540.17	7.11
3F1P	76	13.75	62.14	469.22	5.73
3F1P	68	13.99	36.06	282.33	6.23
3F1P	71	14.02	28.32	194.72	5.00
3F1P	79	14.08	96.38	594.11	9.60
3F1P	66	14.60	51.71	395.97	6.28

3F1P	62	14.83	98.99	715.62	9.56
3F1P	78	14.88	112.82	721.06	10.66
3F1P	73	15.23	95.82	671.28	8.13
3F1P	63	15.83	66.28	450.75	8.16
**PDB-ID**	**Pose Number**	**iRMSD^{B} (Å)**	**Interface Contacts (< 3 Å)**	**Interface Contacts (< 6 Å)**	**Hydrophobic residues on the BSA**
2VXT	10	2.51	139.91	1044.80	8.84
2VXT	12	7.11	92.34	655.40	3.07
2VXT	6	9.17	120.37	865.15	4.99
2VXT	14	10.30	18.33	139.67	3.61
2VXT	7	10.42	2.21	18.67	0.06
2VXT	3	11.20	102.99	723.87	4.69
2VXT	1	11.48	99.53	742.09	2.81
2VXT	13	11.73	43.10	322.51	2.38
2VXT	11	11.75	67.41	476.29	2.76
2VXT	2	12.93	44.11	392.10	2.28
2VXT	4	13.13	149.76	1098.00	8.34
2VXT	8	13.15	94.45	665.63	2.48
2VXT	9	13.53	111.40	881.15	6.10
2VXT	5	15.15	85.71	711.85	8.47
**PDB-ID**	**Pose Number**	**iRMSD^{B} (Å)**	**Interface Contacts (< 3 Å)**	**Interface Contacts (< 6 Å)**	**Hydrophobic residues on the BSA**
3K75	25	3.38	94.04	583.90	6.24
3K75	21	6.86	100.24	616.72	7.29
3K75	32	7.99	73.07	464.93	4.75
3K75	27	8.15	59.28	391.50	4.17
3K75	30	8.48	50.25	320.57	5.19
3K75	33	8.64	90.27	639.05	5.19
3K75	15	8.85	54.93	301.67	6.40
3K75	34	10.48	53.60	369.19	4.87
3K75	28	11.94	106.23	692.37	9.91
3K75	17	12.39	60.62	396.70	10.57
3K75	22	12.46	60.49	367.89	2.76
3K75	24	12.94	145.66	838.25	10.96
3K75	20	12.97	136.49	903.81	15.00
3K75	26	13.33	115.28	856.68	6.74
3K75	29	14.01	174.48	1117.69	12.68
3K75	23	14.50	69.96	533.60	4.38
3K75	31	14.77	97.09	646.96	8.70
3K75	18	15.01	67.89	488.71	4.49
3K75	16	16.81	120.60	877.48	6.09
3K75	19	17.73	166.31	1309.89	7.76
**PDB-ID**	**Pose Number**	**iRMSD^{B} (Å)**	**Interface Contacts (< 3 Å)**	**Interface Contacts (< 6 Å)**	**Hydrophobic residues on the BSA**
4H03	42	2.99	82.03	545.74	6.79
4H03	54	4.80	124.73	986.09	7.13
4H03	40	6.80	140.17	1062.69	13.38
4H03	43	8.92	41.95	399.87	4.39

4H03	38	11.19	107.17	806.77	10.92
4H03	52	12.01	76.36	611.25	8.68
4H03	45	12.66	53.70	392.97	8.13
4H03	44	14.97	95.09	584.82	13.15
4H03	35	15.04	121.22	870.30	9.16
4H03	53	15.38	123.30	902.78	10.83
4H03	39	15.61	145.09	1046.48	11.21
4H03	49	16.18	108.13	647.63	11.00
4H03	50	16.20	141.33	1117.45	15.00
4H03	48	17.45	98.98	571.04	9.98
4H03	36	17.64	92.32	575.94	11.46
4H03	37	18.53	69.90	498.58	8.39
4H03	51	19.20	46.57	402.09	9.93
4H03	47	19.26	80.09	668.53	10.95
4H03	46	21.29	113.85	867.43	9.82
4H03	41	21.74	125.64	742.94	11.54

PDB-ID	Pose Number	iRMSD^{B} (Å)	Interface Contacts (< 3 Å)	Interface Contacts (< 6 Å)	Hydrophobic residues on the BSA
4G6M	55	3.22	157.80	1203.38	9.62
4G6M	59	7.38	59.38	489.53	3.22
4G6M	70	7.94	65.06	323.40	3.50
4G6M	63	9.81	63.51	525.60	3.46
4G6M	60	10.38	33.08	228.00	3.97
4G6M	58	12.52	73.12	633.61	2.60
4G6M	71	12.95	59.53	478.12	3.11
4G6M	67	13.02	10.30	73.37	0.55
4G6M	61	13.46	27.82	199.89	1.52
4G6M	57	13.84	46.50	288.66	2.28
4G6M	73	14.16	97.58	764.15	5.30
4G6M	65	14.33	51.38	336.97	2.70
4G6M	66	14.56	38.42	268.45	1.18
4G6M	64	14.64	95.25	735.38	4.56
4G6M	72	14.84	80.46	524.10	4.00
4G6M	62	14.88	43.28	288.93	5.20
4G6M	74	15.07	48.68	331.86	4.31
4G6M	56	15.41	74.97	639.30	4.39
4G6M	68	15.43	27.40	198.99	4.08
4G6M	69	16.19	54.27	392.39	4.96

Hydrophobic scales

In Table S8 are summarized the hydrophobic scoring of the interfaces, i.e.. the sum of the hydrophobicity of the residues that form the protein-protein interfaces, calculated according to different hydrophobicity scales for the initial poses. Our protocol consisted in individuating the residues at the binding interface and then summing their hydrophobicity. Several hydrophobicity scales were considered: KD [6], WW [7], HH [8], MF[9], TT [10]. Among all the representative poses. the nearest native ones are expected to have the highest value of interface hydrophobicity. Such descriptors rank the nearest native pose in only 1 case out of 8 (3K75). Moreover, the different scales are not in agreement in individuating the most hydrophobic interface along the series of the poses.

***Table S8**: values of the hydrophobicity-based scoring of the protein-protein interfaces, calculated for the initial poses of each PP complex. The poses are ordered according to the iRMSD^B, in ascending order. All the descriptors that identify the most stable pose are highlighted in yellow. Such descriptors rank the nearest-native pose in only 1 case out of 8 (3K75).*

PDB-ID	Pose #	iRMSD^B (Å)	KD scale	WW scale	HH scale	MF scale	TT scale
1JTD	1	1.77	-66.20	-10.67	75.24	64.63	-38.35
1JTD	7	3.88	-48.60	-1.76	60.18	52.77	-24.59
1JTD	9	4.33	-82.20	-18.15	83.71	82.19	-53.18
1JTD	3	8.38	-56.00	-5.72	64.08	56.79	-30.66
1JTD	14	11.40	-25.00	8.23	37.49	33.91	-1.11
1JTD	4	11.51	-62.30	-9.68	68.44	63.11	-35.19
1JTD	19	12.26	-54.40	-2.57	61.81	61.67	-27.76
1JTD	20	12.75	-64.50	-3.64	63.66	64.63	-29.27
1JTD	6	13.96	-86.30	-14.92	82.41	70.06	-51.62
1JTD	18	14.15	-36.70	0.44	40.53	31.24	-16.03
1JTD	8	14.87	-78.90	-9.60	79.49	78.27	-44.03
1JTD	16	14.90	-47.30	0.33	48.80	51.16	-19.38
1JTD	11	15.45	-91.10	-17.53	88.46	78.47	-57.24
1JTD	12	16.03	-68.30	-4.69	63.54	65.61	-32.91
1JTD	10	16.51	-93.60	-16.81	86.76	76.99	-55.99
1JTD	15	16.76	-63.60	-6.82	66.02	68.79	-34.82
1JTD	13	17.24	-91.20	-11.16	84.94	72.52	-48.31
1JTD	5	17.32	-86.00	-10.19	81.64	76.19	-47.52
1JTD	2	17.40	-45.60	-5.84	62.94	53.52	-27.53
1JTD	17	18.01	-76.70	-9.13	71.51	69.36	-42.39
**PDB-ID**	**Pose #**	**iRMSD^B (Å)**	**KD scale**	**WW scale**	**HH scale**	**MF scale**	**TT scale**
2YVJ	24	3.43	-95.90	-28.69	103.05	103.47	-75.99
2YVJ	40	3.86	-78.50	-16.73	78.55	101.30	-54.14
2YVJ	21	5.35	-113.30	-33.18	122.33	146.04	-89.53
2YVJ	36	5.53	-91.10	-25.13	93.70	103.98	-69.95
2YVJ	35	7.49	-73.20	-12.89	71.49	91.39	-49.34
2YVJ	29	7.93	-81.60	-26.81	107.16	115.94	-74.30
2YVJ	30	9.31	-89.70	-23.09	86.81	108.39	-63.43
2YVJ	26	18.04	-41.40	-12.73	48.46	48.74	-30.17
2YVJ	31	18.71	-44.10	-13.67	56.77	62.39	-35.81
2YVJ	38	18.79	-46.30	-16.76	61.34	62.09	-39.95
2YVJ	33	18.86	-44.30	-14.15	56.49	52.10	-33.58
2YVJ	37	18.99	-61.20	-17.09	62.65	83.41	-42.42
2YVJ	34	19.12	-26.10	-9.22	36.98	53.33	-25.52
2YVJ	23	19.58	-66.20	-24.42	69.10	75.21	-51.95
2YVJ	22	19.75	-71.90	-27.44	84.45	94.75	-59.95
2YVJ	32	19.76	-54.10	-21.83	70.52	83.31	-48.40
2YVJ	28	19.85	-35.70	-15.58	57.26	68.33	-33.65
2YVJ	39	20.09	-34.10	-13.79	53.84	54.54	-37.58
2YVJ	25	20.48	-28.50	-11.40	59.43	48.41	-35.84
2YVJ	27	21.37	-35.20	-15.30	43.66	48.93	-32.47
**PDB-ID**	**Pose #**	**iRMSD^B (Å)**	**KD scale**	**WW scale**	**HH scale**	**MF scale**	**TT scale**

3PC8	41	1.02	-29.40	-10.02	43.35	45.08	-23.87
3PC8	52	4.84	-30.90	-16.55	63.21	67.57	-33.61
3PC8	55	9.70	-33.90	-9.40	40.77	70.34	-23.25
3PC8	49	9.98	-28.20	-8.66	45.54	45.39	-23.15
3PC8	45	10.30	-28.40	-9.83	42.76	51.93	-23.64
3PC8	56	10.61	-28.50	-15.39	41.89	61.01	-29.05
3PC8	50	10.81	-52.40	-17.15	53.46	61.07	-38.38
3PC8	51	11.42	-26.20	-11.66	42.98	34.84	-21.42
3PC8	43	11.79	-47.40	-19.20	65.07	75.18	-42.37
3PC8	47	11.93	-42.60	-9.08	45.84	47.32	-26.03
3PC8	59	12.06	-22.00	-6.11	28.05	32.98	-14.12
3PC8	60	12.93	-33.20	-10.88	53.98	51.99	-26.13
3PC8	42	13.05	-43.00	-12.82	43.74	39.79	-30.30
3PC8	44	13.82	-36.00	-10.99	43.89	38.68	-26.41
3PC8	46	14.05	-38.90	-13.30	40.12	26.66	-28.15
3PC8	57	14.29	-34.40	-9.76	41.95	37.59	-23.82
3PC8	53	14.40	-38.40	-9.58	41.94	36.06	-25.53
3PC8	58	14.44	-42.90	-12.82	52.75	52.49	-30.95
3PC8	48	14.45	-31.80	-11.61	52.39	49.06	-27.60
3PC8	54	14.46	-30.90	-6.76	39.72	26.77	-19.66
**PDB-ID**	**Pose #**	**iRMSD^B (Å)**	**KD scale**	**WW scale**	**HH scale**	**MF scale**	**TT scale**
3F1P	72	2.95	-38.60	-10.89	67.93	65.47	-30.52
3F1P	69	7.89	-37.10	-11.07	66.82	77.87	-29.33
3F1P	74	8.64	-43.70	-9.36	59.26	55.08	-28.95
3F1P	67	9.01	-25.70	-7.75	60.47	52.47	-22.18
3F1P	61	10.01	-41.20	-8.23	52.74	53.85	-28.87
3F1P	70	11.13	-37.40	-17.57	57.94	58.55	-32.54
3F1P	65	11.29	-34.60	-10.23	48.82	48.49	-27.47
3F1P	80	12.06	-50.40	-12.85	57.73	58.22	-32.61
3F1P	64	13.28	-45.90	-10.31	61.90	55.56	-34.03
3F1P	77	13.39	-59.50	-10.49	56.03	63.79	-35.78
3F1P	75	13.69	-57.00	-15.52	65.90	74.59	-43.62
3F1P	76	13.75	-60.60	-17.10	70.88	72.47	-42.04
3F1P	68	13.99	-57.20	-14.98	57.07	60.04	-40.05
3F1P	71	14.02	-20.80	-32.80	33.63	35.27	-11.37
3F1P	79	14.08	-26.90	-10.47	59.11	53.55	-24.48
3F1P	66	14.60	-43.80	-9.94	56.71	60.24	-29.95
3F1P	62	14.83	-30.80	-12.03	61.30	59.68	-27.41
3F1P	78	14.88	-42.10	-11.81	58.15	55.00	-33.73
3F1P	73	15.23	-45.60	-87.10	57.22	62.13	-30.12
3F1P	63	15.83	-26.30	-10.76	55.78	50.32	-24.94
**PDB-ID**	**Pose #**	**iRMSD^B (Å)**	**KD scale**	**WW scale**	**HH scale**	**MF scale**	**TT scale**
2VXT	10	2.51	-88.70	-14.96	86.67	90.42	-55.28
2VXT	12	7.11	-80.10	-9.70	83.43	83.13	-50.08
2VXT	6	9.17	-86.80	-17.49	82.94	84.86	-57.27
2VXT	14	10.30	-44.80	-1.66	43.54	43.72	-26.53
2VXT	7	10.42	-80.00	-9.20	73.73	78.69	-50.32
2VXT	3	11.20	-100.40	-20.35	95.68	96.29	-69.06
2VXT	1	11.48	-99.60	-12.66	87.44	98.06	-63.32

2VXT	13	11.73	-79.40	-6.40	72.62	71.68	-46.02
2VXT	11	11.75	-57.70	-5.20	55.99	55.88	-35.16
2VXT	2	12.93	-60.20	-5.34	67.23	76.58	-40.29
2VXT	4	13.13	-84.00	-17.95	85.49	96.37	-60.55
2VXT	8	13.15	-58.00	-2.73	63.02	67.78	-35.87
2VXT	9	13.53	-82.50	-17.25	84.45	98.99	-60.80
2VXT	5	15.15	-65.30	-11.24	67.12	67.71	-45.31
**PDB-ID**	**Pose #**	**iRMSD[B] (Å)**	**KD scale**	**WW scale**	**HH scale**	**MF scale**	**TT scale**
3K75	25	3.38	-7.40	-8.86	44.05	29.56	-17.26
3K75	21	6.86	-105.80	-30.75	107.90	107.30	-77.62
3K75	32	7.99	-47.20	-17.81	67.57	63.21	-43.26
3K75	27	8.15	-55.70	-17.14	65.82	55.36	-47.06
3K75	30	8.48	-92.60	-25.74	89.31	82.98	-63.41
3K75	33	8.64	-45.40	-8.67	55.51	50.12	-32.57
3K75	15	8.85	-51.40	-24.31	82.16	92.23	-49.71
3K75	34	10.48	-54.50	-16.59	65.95	66.64	-45.12
3K75	28	11.94	-73.70	-24.14	85.93	102.14	-55.60
3K75	17	12.39	-77.00	-22.74	82.09	89.51	-57.84
3K75	22	12.46	-40.50	-10.50	46.57	41.97	-31.17
3K75	24	12.94	-105.70	-34.01	121.66	135.18	-80.89
3K75	20	12.97	-110.40	-34.01	119.53	145.47	-83.91
3K75	26	13.33	-134.80	-37.98	132.61	166.06	-99.72
3K75	29	14.01	-102.60	-32.33	110.31	131.74	-79.35
3K75	23	14.50	-62.80	-18.17	58.99	64.05	-42.82
3K75	31	14.77	-64.50	-18.76	63.07	68.02	-46.46
3K75	18	15.01	-58.60	-22.09	76.91	74.82	-52.35
3K75	16	16.81	-119.60	-36.49	121.31	135.64	-90.47
3K75	19	17.73	-155.00	-43.65	145.12	161.09	-113.64
**PDB-ID**	**Pose #**	**iRMSD[B] (Å)**	**KD scale**	**WW scale**	**HH scale**	**MF scale**	**TT scale**
4H03	42	2.99	-51.50	-17.74	70.21	60.58	-37.60
4H03	54	4.80	-60.90	-21.73	82.58	87.79	-49.81
4H03	40	6.80	-49.00	-14.79	74.76	79.90	-40.14
4H03	43	8.92	-59.00	-19.08	78.61	67.76	-46.10
4H03	38	11.19	-52.20	-20.27	82.40	92.00	-49.40
4H03	52	12.01	-56.30	-18.44	78.80	83.97	-46.87
4H03	45	12.66	-55.40	-15.36	62.67	65.94	-40.44
4H03	44	14.97	-30.70	-7.14	43.91	48.71	-22.62
4H03	35	15.04	-49.60	-17.82	74.42	77.62	-44.66
4H03	53	15.38	-43.90	-12.06	63.67	79.40	-37.63
4H03	39	15.61	-91.60	-26.23	118.92	132.32	-69.92
4H03	49	16.18	-41.50	-12.64	68.76	76.91	-36.98
4H03	50	16.20	-66.50	-17.49	78.90	98.29	-49.60
4H03	48	17.45	-51.30	-10.39	60.07	77.02	-36.55
4H03	36	17.64	-28.10	-8.77	57.09	60.85	-21.46
4H03	37	18.53	-46.70	-11.08	51.96	61.53	-33.00
4H03	51	19.20	-34.90	-19.00	66.99	60.46	-38.40
4H03	47	19.26	-48.10	-13.89	76.03	88.27	-37.91
4H03	46	21.29	-42.00	-10.40	63.66	55.42	-32.98
4H03	41	21.74	-66.70	-20.21	80.96	99.57	-51.00

PDB-ID	Pose #	iRMSD[B] (Å)	KD scale	WW scale	HH scale	MF scale	TT scale
4G6M	55	3.22	-85.40	-11.95	83.34	78.70	-56.40
4G6M	59	7.38	-73.10	-7.84	73.31	73.78	-47.12
4G6M	70	7.94	-70.00	-9.23	65.39	74.61	-44.06
4G6M	63	9.81	-64.50	-3.64	66.40	70.28	-37.29
4G6M	60	10.38	-77.00	-10.06	64.02	67.38	-49.44
4G6M	58	12.52	-58.20	-8.26	61.10	70.95	-40.17
4G6M	71	12.95	-57.10	-5.06	55.46	58.28	-36.00
4G6M	67	13.02	-64.30	-11.86	76.24	70.78	-45.84
4G6M	61	13.46	-61.80	-6.35	52.07	54.46	-37.00
4G6M	57	13.84	-54.70	-2.97	60.05	77.05	-28.44
4G6M	73	14.16	-48.00	-2.33	52.43	59.05	-25.83
4G6M	65	14.33	-41.30	-3.63	45.73	51.67	-22.60
4G6M	66	14.56	-62.40	-8.98	61.03	64.45	-40.02
4G6M	64	14.64	-73.90	-10.32	71.03	74.60	-44.32
4G6M	72	14.84	-71.90	-11.52	64.05	73.80	-44.38
4G6M	62	14.88	-53.20	-6.50	59.32	71.35	-32.96
4G6M	74	15.07	-71.50	-10.34	72.11	95.38	-43.34
4G6M	56	15.41	-79.20	-10.35	73.72	75.08	-44.13
4G6M	68	15.43	-70.10	-8.96	65.80	80.06	-41.15
4G6M	69	16.19	-71.90	-6.64	62.84	77.51	-38.28

Comparison of the descriptors along the 5ns SMD trajectories

In Tables from S9 to S12 are summarized the average values of the descriptors (iRMSD, BSA, HBS and HBS/iRMSD) calculated in the first 5 ns of the 3 SMD trajectories for each pose. The calculation of each descriptor was carried out considering a frame each 100 ps. Every descriptor was first averaged on each of the three trajectories, then averaged of the three trajectories. The calculation of the iRMSD the following protocol has been used:

1) The residues at the interface between the two proteins were found using the pymol script *InterfaceResidues* (http://www.protein.osaka-u.ac.jp/rcsfp/supracryst/suzuki/jpxtal/Katsutani/en/interface.php). setting 1 Å as cutoff.

2) The iRMSD was then calculated along the trajectories considering all the atoms of these residues.

Table S9: *average values of iRMSD calculated for each pose along three different SMD trajectories for the first 5ns (iRMSD 1st traj, iRMSD 2nd traj and iRMSD 3rd traj), and iRMSD values averaged on the three trajectories (Average iRMSD). The pose number highlighted in yellow represents the nearest native poses, having the lowest value of iRMSD^B (see Table S1). The lowest values of iRMSD that identify the most stable pose are highlighted in yellow.*

PDB-ID	Pose Number	iRMSD 1st traj (Å)	iRMSD 2nd traj (Å)	iRMSD 3rd traj (Å)	Average iRMSD (Å)
1JTD	1 (native)	3.332	5.495	3.651	4.160
1JTD	2	4.417	5.216	6.683	5.439
1JTD	3	6.970	3.052	7.127	5.717
1JTD	4	13.065	11.895	9.315	11.425
1JTD	5	4.912	6.260	3.695	4.955
1JTD	6	6.861	2.941	5.456	5.086
1JTD	7	4.696	4.774	3.958	4.476
1JTD	8	6.854	2.695	4.907	4.819
1JTD	9	4.435	13.781	3.431	7.216
1JTD	10	3.208	3.375	3.477	3.353
1JTD	11	4.900	3.836	26.378	11.705
1JTD	12	7.185	8.847	7.547	7.860
1JTD	13	4.447	3.135	2.916	3.499
1JTD	14	2.768	6.272	2.359	3.800
1JTD	15	3.911	3.097	3.275	3.428
1JTD	16	2.966	3.973	2.347	3.095
1JTD	17	6.636	4.853	4.830	5.440
1JTD	18	9.509	3.923	4.443	5.958
1JTD	19	3.096	5.535	3.037	3.889
1JTD	20	2.855	3.623	4.052	3.510
**PDB-ID**	**Pose Number**	**iRMSD 1st traj (Å)**	**iRMSD 2nd traj (Å)**	**iRMSD 3rd traj (Å)**	**Average iRMSD (Å)**
2YVJ	21	3.354	4.166	2.921	3.480
2YVJ	22	3.367	2.908	4.856	3.710
2YVJ	23	2.722	3.633	5.741	4.032
2YVJ	24 (native)	3.431	3.485	3.555	3.490
2YVJ	25	3.140	3.171	2.345	2.886
2YVJ	26	6.046	3.174	3.867	4.363
2YVJ	27	3.534	7.174	3.844	4.850
2YVJ	28	6.668	7.660	6.089	6.806
2YVJ	29	3.461	3.624	3.918	3.668
2YVJ	30	4.471	3.434	4.402	4.102
2YVJ	31	2.802	3.650	8.549	5.000
2YVJ	32	4.712	9.713	3.106	5.844
2YVJ	33	5.680	4.143	4.431	4.751
2YVJ	34	3.499	3.323	10.720	5.847
2YVJ	35	5.069	2.639	3.029	3.579
2YVJ	36	3.217	3.205	3.368	3.263
2YVJ	37	3.798	2.864	3.955	3.539
2YVJ	38	4.614	5.348	4.911	4.958
2YVJ	39	4.683	5.307	3.681	4.557

2YVJ	40	4.325	4.661	4.122	4.370
**PDB-ID**	**Pose Number**	**iRMSD 1st traj (Å)**	**iRMSD 2nd traj (Å)**	**iRMSD 3rd traj (Å)**	**Average iRMSD (Å)**
3PC8	41 (native)	1.771	2.113	2.113	1.999
3PC8	42	3.570	3.299	3.593	3.487
3PC8	43	6.515	3.723	4.431	4.890
3PC8	44	4.096	4.222	4.777	4.365
3PC8	45	4.420	3.664	3.782	3.956
3PC8	46	8.813	4.836	6.167	6.605
3PC8	47	3.284	4.536	3.837	3.886
3PC8	48	2.344	3.540	2.517	2.800
3PC8	49	5.642	4.157	4.245	4.681
3PC8	50	2.307	2.512	2.550	2.456
3PC8	51	4.756	4.389	4.565	4.570
3PC8	52	4.839	4.062	4.689	4.530
3PC8	53	4.036	3.704	3.471	3.737
3PC8	54	3.679	5.021	2.765	3.822
3PC8	55	3.235	4.590	7.146	4.990
3PC8	56	2.829	3.118	3.445	3.131
3PC8	57	2.525	2.576	2.333	2.478
3PC8	58	4.160	4.560	4.462	4.394
3PC8	59	3.895	5.515	2.792	4.068
3PC8	60	5.726	2.866	6.830	5.141
**PDB-ID**	**Pose Number**	**iRMSD 1st traj (Å)**	**iRMSD 2nd traj (Å)**	**iRMSD 3rd traj (Å)**	**Average iRMSD (Å)**
3F1P	61	5.681	2.943	7.526	5.383
3F1P	62	3.609	5.640	6.363	5.204
3F1P	63	4.060	4.445	4.943	4.483
3F1P	64	5.904	4.153	3.700	4.586
3F1P	65	2.990	4.252	4.003	3.748
3F1P	66	3.937	3.733	3.884	3.851
3F1P	67	4.194	4.667	3.502	4.121
3F1P	68	3.898	4.425	6.752	5.025
3F1P	69	4.293	6.085	4.496	4.958
3F1P	70	3.633	3.869	4.065	3.856
3F1P	71	7.408	5.600	5.304	6.104
3F1P	72 (native)	3.475	3.769	3.072	3.438
3F1P	73	3.892	4.678	4.484	4.352
3F1P	74	3.290	3.588	3.138	3.339
3F1P	75	5.141	4.098	3.549	4.262
3F1P	76	5.555	8.402	8.365	7.440
3F1P	77	4.436	4.404	4.783	4.541
3F1P	78	2.740	2.932	2.972	2.881
3F1P	79	3.306	3.642	5.527	4.158
3F1P	80	4.673	3.673	5.367	4.571
**PDB-ID**	**Pose Number**	**iRMSD 1st traj (Å)**	**iRMSD 2nd traj (Å)**	**iRMSD 3rd traj (Å)**	**Average iRMSD (Å)**
2VXT	1	3.762	3.296	3.199	3.419

2VXT	2	5.051	7.028	3.407	5.162
2VXT	3	4.872	4.545	4.814	4.744
2VXT	4	2.462	2.834	4.529	3.275
2VXT	5	3.238	6.096	3.401	4.245
2VXT	6	3.160	3.507	4.938	3.869
2VXT	7	17.412	16.517	7.211	13.713
2VXT	8	3.040	3.086	2.880	3.002
2VXT	9	2.880	3.365	2.708	2.984
2VXT	10 (native)	3.261	3.296	3.362	3.306
2VXT	11	4.943	3.670	5.868	4.827
2VXT	12	3.368	4.788	3.445	3.867
2VXT	13	4.048	4.588	5.887	4.841
2VXT	14	7.261	6.205	5.655	6.373
**PDB-ID**	**Pose Number**	**iRMSD 1st traj (Å)**	**iRMSD 2nd traj (Å)**	**iRMSD 3rd traj (Å)**	**Average iRMSD (Å)**
3K75	15	4.143	3.359	4.437	3.980
3K75	16	3.023	4.165	2.924	3.370
3K75	17	3.418	2.365	2.442	2.742
3K75	18	5.677	2.509	2.450	3.545
3K75	19	4.237	3.974	3.070	3.760
3K75	20	4.067	2.932	3.616	3.538
3K75	21	4.453	3.882	4.103	4.146
3K75	22	2.138	2.174	2.657	2.323
3K75	23	2.912	3.675	3.605	3.397
3K75	24	4.427	3.447	3.443	3.772
3K75	25 (native)	2.242	2.664	2.752	2.553
3K75	26	4.844	3.746	4.193	4.261
3K75	27	2.983	3.112	4.196	3.430
3K75	28	2.979	2.664	3.199	2.947
3K75	29	2.959	7.359	4.348	4.889
3K75	30	12.797	3.904	4.630	7.111
3K75	31	3.106	3.437	3.827	3.457
3K75	32	4.262	4.314	4.044	4.207
3K75	33	2.352	3.565	2.626	2.848
3K75	34	5.278	13.475	4.756	7.837
**PDB-ID**	**Pose Number**	**iRMSD 1st traj (Å)**	**iRMSD 2nd traj (Å)**	**iRMSD 3rd traj (Å)**	**Average iRMSD (Å)**
4H03	35	3.771	3.918	3.062	3.584
4H03	36	3.356	5.212	3.778	4.115
4H03	37	3.929	3.888	4.364	4.060
4H03	38	3.462	3.877	4.019	3.786
4H03	39	4.151	4.936	4.397	4.495
4H03	40	2.763	3.392	2.703	2.953
4H03	41	5.735	2.970	3.061	3.922
4H03	42 (native)	3.959	3.119	3.437	3.505
4H03	43	4.522	6.651	5.249	5.474
4H03	44	4.630	3.893	4.456	4.326
4H03	45	4.123	5.969	4.100	4.731
4H03	46	2.995	6.856	2.947	4.266

4H03	47	3.675	2.941	3.587	3.401
4H03	48	4.216	2.866	3.174	3.419
4H03	49	3.254	3.099	2.477	2.943
4H03	50	3.559	3.940	4.323	3.941
4H03	51	3.987	5.086	3.337	4.137
4H03	52	5.184	7.463	5.929	6.192
4H03	53	3.221	3.814	3.145	3.393
4H03	54	2.758	4.618	2.720	3.365

PDB-ID	Pose Number	iRMSD 1st traj (Å)	iRMSD 2nd traj (Å)	iRMSD 3rd traj (Å)	Average iRMSD (Å)
4G6M	55 (native)	1.924	2.636	1.980	2.180
4G6M	56	4.644	4.926	6.072	5.214
4G6M	57	6.113	6.299	5.281	5.898
4G6M	58	3.324	3.763	2.974	3.353
4G6M	59	4.394	3.490	4.687	4.190
4G6M	60	5.672	5.988	6.944	6.201
4G6M	61	4.342	6.405	7.705	6.151
4G6M	62	7.110	6.000	6.404	6.505
4G6M	63	3.054	3.984	3.213	3.417
4G6M	64	3.194	4.313	2.919	3.476
4G6M	65	3.608	4.079	2.979	3.555
4G6M	66	6.439	5.335	4.649	5.474
4G6M	67	14.091	6.134	7.469	9.231
4G6M	68	6.114	4.307	9.626	6.682
4G6M	69	4.003	3.648	4.644	4.098
4G6M	70	7.188	7.089	4.204	6.160
4G6M	71	3.535	2.167	4.144	3.282
4G6M	72	4.417	4.599	4.934	4.650
4G6M	73	2.652	3.111	3.009	2.924
4G6M	74	6.455	2.378	6.218	5.017

Table S10: *average values of BSA calculated for each pose along three different SMD trajectories for the first 5ns (BSA 1st traj, BSA 2nd traj and BSA 3rd traj), and BSA values averaged on the three trajectories (Average BSA). The pose number highlighted in yellow represents the nearest native poses, having the lowest value of iRMSD[B] (see Table S1). The highest values of BSA that identify the most stable pose are highlighted in yellow.*

PDB-ID	Pose Number	BSA 1st traj (Å^2)	BSA 2nd traj (Å^2)	BSA 3rd traj (Å^2)	Average BSA (Å^2)
1JTD	1 (native)	955.813	665.133	585.940	735.629
1JTD	2	535.872	735.990	478.625	583.496
1JTD	3	242.055	444.433	821.119	502.535
1JTD	4	325.544	241.241	179.547	248.778
1JTD	5	647.527	349.582	582.379	526.496
1JTD	6	645.149	536.930	521.322	567.800
1JTD	7	686.678	491.582	771.508	649.923
1JTD	8	422.841	657.782	631.778	570.800
1JTD	9	629.828	119.160	861.132	536.707

1JTD	10	746.242	760.317	839.707	782.089
1JTD	11	442.851	850.310	51.086	448.082
1JTD	12	395.249	426.585	319.254	380.363
1JTD	13	621.644	843.922	1114.718	860.095
1JTD	14	838.039	355.604	964.260	719.301
1JTD	15	657.106	548.183	823.579	676.289
1JTD	16	572.331	634.861	652.828	620.006
1JTD	17	784.629	439.175	638.589	620.798
1JTD	18	213.919	699.288	421.610	444.939
1JTD	19	620.177	225.449	613.986	486.537
1JTD	20	584.644	577.946	633.532	598.707
**PDB-ID**	**Pose Number**	**BSA 1st traj (Å^2)**	**BSA 2nd traj (Å^2)**	**BSA 3rd traj (Å^2)**	**Average BSA (Å^2)**
2YVJ	21	765.798	792.297	1005.065	854.387
2YVJ	22	936.309	1047.690	526.341	836.780
2YVJ	23	721.034	868.705	389.307	659.682
2YVJ	24 (native)	936.238	615.212	810.405	787.285
2YVJ	25	636.388	620.835	617.564	624.929
2YVJ	26	399.726	419.682	561.756	460.388
2YVJ	27	472.269	347.638	582.943	467.617
2YVJ	28	336.106	565.020	333.534	411.554
2YVJ	29	970.924	1129.660	848.228	982.937
2YVJ	30	857.222	656.430	511.509	675.054
2YVJ	31	712.404	843.724	523.628	693.252
2YVJ	32	498.239	382.269	625.673	502.061
2YVJ	33	359.697	470.457	403.540	411.231
2YVJ	34	421.376	416.708	291.215	376.433
2YVJ	35	610.360	1113.995	859.292	861.215
2YVJ	36	670.718	1015.871	1114.771	933.787
2YVJ	37	581.188	853.726	548.946	661.286
2YVJ	38	549.586	376.331	376.295	434.071
2YVJ	39	425.160	690.987	610.316	575.488
2YVJ	40	726.319	906.185	766.426	799.644
**PDB-ID**	**Pose Number**	**BSA 1st traj (Å^2)**	**BSA 2nd traj (Å^2)**	**BSA 3rd traj (Å^2)**	**Average BSA (Å^2)**
3PC8	41 (native)	649.552	656.474	646.555	650.860
3PC8	42	635.674	549.248	607.072	597.331
3PC8	43	544.529	489.205	521.498	518.411
3PC8	44	603.382	419.887	446.839	490.036
3PC8	45	545.872	699.185	529.527	591.528
3PC8	46	370.659	337.092	365.172	357.641
3PC8	47	573.431	494.340	667.162	578.311
3PC8	48	715.185	524.207	739.138	659.510
3PC8	49	687.105	682.759	722.760	697.541
3PC8	50	690.268	626.842	728.740	681.950
3PC8	51	434.127	332.600	427.592	398.106
3PC8	52	707.731	961.029	496.705	721.822
3PC8	53	452.515	925.838	743.555	707.303
3PC8	54	390.120	413.168	592.171	465.153
3PC8	55	460.341	384.434	373.498	406.091
3PC8	56	536.486	540.364	517.461	531.437

3PC8	57	689.306	660.964	630.824	660.365
3PC8	58	793.045	626.398	701.483	706.975
3PC8	59	415.814	322.435	462.276	400.175
3PC8	60	599.484	806.113	531.821	645.806
**PDB-ID**	**Pose Number**	**BSA 1st traj (Å^2)**	**BSA 2nd traj (Å^2)**	**BSA 3rd traj (Å^2)**	**Average BSA (Å^2)**
3F1P	61	536.560	645.918	305.258	495.912
3F1P	62	788.903	435.109	600.762	608.258
3F1P	63	571.419	643.583	575.461	596.821
3F1P	64	565.111	690.080	775.972	677.054
3F1P	65	809.874	911.431	709.790	810.365
3F1P	66	647.915	730.453	702.905	693.757
3F1P	67	958.291	860.458	965.529	928.093
3F1P	68	520.479	696.558	615.161	610.733
3F1P	69	698.830	794.544	745.547	746.307
3F1P	70	552.754	594.114	553.735	566.868
3F1P	71	293.228	406.048	669.622	456.299
3F1P	72 (native)	1163.294	1114.286	1128.627	1135.400
3F1P	73	762.904	850.805	808.692	807.467
3F1P	74	546.831	656.701	580.807	594.780
3F1P	75	985.611	931.002	965.584	960.732
3F1P	76	500.470	396.180	459.689	452.113
3F1P	77	878.471	591.140	766.626	745.412
3F1P	78	1137.329	981.056	1073.770	1064.050
3F1P	79	634.231	763.359	707.241	701.610
3F1P	80	701.696	797.319	503.868	667.628
**PDB-ID**	**Pose Number**	**BSA 1st traj (Å^2)**	**BSA 2nd traj (Å^2)**	**BSA 3rd traj (Å^2)**	**Average BSA (Å^2)**
2VXT	1	610.937	947.545	805.725	788.069
2VXT	2	367.398	350.205	527.523	415.042
2VXT	3	734.578	570.340	1034.605	779.841
2VXT	4	1020.502	719.930	655.283	798.572
2VXT	5	759.993	475.481	860.390	698.621
2VXT	6	713.465	770.819	359.095	614.460
2VXT	7	56.419	59.518	971.477	362.471
2VXT	8	766.183	927.919	843.316	845.806
2VXT	9	892.544	1188.567	1186.472	1089.190
2VXT	10 (native)	1099.783	1215.846	1031.321	1115.650
2VXT	11	659.365	676.999	374.009	570.124
2VXT	12	721.720	612.668	712.388	682.259
2VXT	13	810.209	483.778	562.561	618.849
2VXT	14	286.921	458.275	705.179	483.458
**PDB-ID**	**Pose Number**	**BSA 1st traj (Å^2)**	**BSA 2nd traj (Å^2)**	**BSA 3rd traj (Å^2)**	**Average BSA (Å^2)**
3K75	15	643.127	661.131	692.336	665.531
3K75	16	904.281	1040.025	1027.953	990.753
3K75	17	914.573	1244.387	1076.585	1078.510
3K75	18	502.374	700.913	752.365	651.884
3K75	19	1233.701	1108.679	1211.774	1184.720
3K75	20	1177.124	1271.471	794.202	1080.930
3K75	21	816.881	814.154	905.782	845.606

3K75	22	432.111	597.579	544.774	524.821
3K75	23	559.588	747.720	443.956	583.755
3K75	24	1193.683	886.731	1232.839	1104.420
3K75	25 (native)	636.161	600.126	577.406	604.564
3K75	26	858.397	1178.223	1024.975	1020.530
3K75	27	409.931	504.823	561.126	491.960
3K75	28	901.676	768.866	742.751	804.431
3K75	29	1166.747	759.871	621.063	849.227
3K75	30	541.418	979.397	471.566	664.127
3K75	31	957.929	950.734	851.747	920.137
3K75	32	416.713	566.286	653.096	545.365
3K75	33	603.706	876.510	698.399	726.205
3K75	34	587.296	157.068	550.216	431.527
**PDB-ID**	**Pose Number**	**BSA 1st traj ($Å^2$)**	**BSA 2nd traj ($Å^2$)**	**BSA 3rd traj ($Å^2$)**	**Average BSA ($Å^2$)**
4H03	35	905.400	1037.096	841.538	928.011
4H03	36	737.632	785.146	1156.682	893.153
4H03	37	750.154	792.622	1008.250	850.342
4H03	38	1012.978	723.948	734.300	823.742
4H03	39	970.354	837.690	1283.116	1030.390
4H03	40	850.602	812.310	769.018	810.643
4H03	41	1076.716	1279.370	969.332	1108.470
4H03	42 (native)	503.364	965.672	783.162	750.733
4H03	43	509.662	589.584	394.774	498.007
4H03	44	909.360	896.022	913.620	906.334
4H03	45	483.352	884.632	644.728	670.904
4H03	46	920.160	452.826	592.570	655.185
4H03	47	858.644	940.272	844.696	881.204
4H03	48	693.206	768.438	804.766	755.470
4H03	49	983.008	574.596	893.382	816.995
4H03	50	1251.552	767.452	598.448	872.484
4H03	51	840.478	456.718	606.986	634.727
4H03	52	726.020	576.668	429.884	577.524
4H03	53	1063.368	774.490	1017.270	951.709
4H03	54	893.610	496.974	887.404	759.329
**PDB-ID**	**Pose Number**	**BSA 1st traj ($Å^2$)**	**BSA 2nd traj ($Å^2$)**	**BSA 3rd traj ($Å^2$)**	**Average BSA ($Å^2$)**
4G6M	55 (native)	1402.697	722.880	1222.887	1116.150
4G6M	56	553.491	522.645	644.300	573.479
4G6M	57	614.682	417.751	642.544	558.326
4G6M	58	479.564	497.054	559.729	512.116
4G6M	59	490.112	697.804	511.583	566.500
4G6M	60	474.317	251.223	443.380	389.640
4G6M	61	290.122	266.024	485.097	347.081
4G6M	62	532.560	519.996	350.250	467.602
4G6M	63	572.953	248.530	559.426	460.303
4G6M	64	941.711	551.855	794.468	762.678
4G6M	65	609.190	404.536	415.414	476.380
4G6M	66	221.801	378.184	229.522	276.502
4G6M	67	152.936	324.862	240.839	239.546
4G6M	68	536.998	458.199	317.348	437.515

4G6M	69	592.156	647.048	497.245	578.816
4G6M	70	448.114	163.684	356.956	322.918
4G6M	71	448.363	467.507	378.396	431.422
4G6M	72	591.350	324.364	311.345	409.020
4G6M	73	768.724	506.129	628.781	634.545
4G6M	74	501.481	641.200	641.724	594.801

Table S11: *average values of HBS calculated for each pose along three different SMD trajectories for the first 5ns (HBS 1st traj, HBS 2nd traj and HBS 3rd traj), and HBS values averaged on the three trajectories (Average HBS). The pose number highlighted in yellow represents the nearest native poses, having the lowest value of iRMSD[B] (see Table S1). The highest values of HBS that identify the most stable pose are highlighted in yellow*

PDB-ID	Pose Number	HBS 1st traj	HBS 2nd traj	HBS 3rd traj	Average HBS
1JTD	1 (native)	15.480	11.340	8.560	11.793
1JTD	2	7.920	9.500	9.660	9.027
1JTD	3	0.840	2.980	14.480	6.100
1JTD	4	4.300	2.380	2.240	2.973
1JTD	5	4.380	1.940	3.720	3.347
1JTD	6	2.840	5.980	3.380	4.067
1JTD	7	9.960	5.940	10.560	8.820
1JTD	8	2.720	5.200	6.440	4.787
1JTD	9	13.260	1.720	8.520	7.833
1JTD	10	5.340	9.000	5.380	6.573
1JTD	11	7.540	9.620	0.400	5.853
1JTD	12	2.840	4.900	2.160	3.300
1JTD	13	4.820	6.940	10.920	7.560
1JTD	14	8.520	2.920	9.820	7.087
1JTD	15	8.240	4.800	9.560	7.533
1JTD	16	6.020	5.100	5.460	5.527
1JTD	17	5.700	2.780	4.420	4.300
1JTD	18	1.420	8.440	3.500	4.453
1JTD	19	7.980	2.240	8.080	6.100
1JTD	20	9.780	7.760	9.840	9.127
**PDB-ID**	**Pose Number**	**HBS 1st traj**	**HBS 2nd traj**	**HBS 3rd traj**	**Average HBS**
2YVJ	21	10.600	12.220	15.480	12.767
2YVJ	22	14.060	18.680	7.620	13.453
2YVJ	23	10.100	12.320	3.360	8.593
2YVJ	24 (native)	19.460	7.860	13.420	13.580
2YVJ	25	16.660	19.980	13.900	16.847
2YVJ	26	12.620	18.100	18.360	16.360
2YVJ	27	3.420	5.000	7.040	5.153
2YVJ	28	6.840	10.340	5.560	7.580
2YVJ	29	20.420	20.580	13.520	18.173
2YVJ	30	14.800	7.960	5.700	9.487
2YVJ	31	10.560	13.140	12.620	12.107
2YVJ	32	10.200	5.760	11.260	9.073

2YVJ	33	5.980	5.040	6.280	5.767
2YVJ	34	7.480	12.580	3.140	7.733
2YVJ	35	10.420	14.200	8.380	11.000
2YVJ	36	10.340	14.240	12.920	12.500
2YVJ	37	10.140	13.900	6.880	10.307
2YVJ	38	6.560	5.340	4.740	5.547
2YVJ	39	7.720	15.060	12.160	11.647
2YVJ	40	11.740	13.720	15.120	13.527
**PDB-ID**	**Pose Number**	**HBS 1st traj**	**HBS 2nd traj**	**HBS 3rd traj**	**Average HBS**
3PC8	41 (native)	18.140	16.060	15.880	16.693
3PC8	42	10.680	6.840	8.140	8.553
3PC8	43	9.240	12.900	11.680	11.273
3PC8	44	6.460	4.420	5.040	5.307
3PC8	45	7.240	9.200	6.000	7.480
3PC8	46	4.460	2.320	4.240	3.673
3PC8	47	12.120	6.140	12.120	10.127
3PC8	48	15.020	11.540	14.420	13.660
3PC8	49	14.320	17.360	18.220	16.633
3PC8	50	12.740	8.420	13.640	11.600
3PC8	51	5.180	4.020	4.580	4.593
3PC8	52	17.520	25.960	9.820	17.767
3PC8	53	5.540	15.720	12.080	11.113
3PC8	54	3.460	4.260	8.120	5.280
3PC8	55	5.700	5.720	7.580	6.333
3PC8	56	9.300	11.540	11.240	10.693
3PC8	57	14.380	13.120	13.360	13.620
3PC8	58	13.160	8.640	9.360	10.387
3PC8	59	2.800	3.060	3.500	3.120
3PC8	60	15.880	18.440	12.760	15.693
**PDB-ID**	**Pose Number**	**HBS 1st traj**	**HBS 2nd traj**	**HBS 3rd traj**	**Average HBS**
3F1P	61	11.660	15.180	4.860	10.567
3F1P	62	16.780	7.820	14.180	12.927
3F1P	63	11.340	12.060	10.440	11.280
3F1P	64	12.560	11.800	13.900	12.753
3F1P	65	11.900	16.640	14.040	14.193
3F1P	66	11.520	12.040	11.500	11.687
3F1P	67	28.640	21.760	28.120	26.173
3F1P	68	8.260	10.580	10.120	9.653
3F1P	69	15.780	22.180	17.900	18.620
3F1P	70	12.400	12.160	9.380	11.313
3F1P	71	6.340	11.560	15.420	11.107
3F1P	72 (native)	27.960	26.180	30.980	28.373
3F1P	73	12.820	15.120	15.460	14.467
3F1P	74	10.200	14.880	11.920	12.333
3F1P	75	15.940	16.260	13.760	15.320
3F1P	76	11.320	5.800	9.860	8.993
3F1P	77	12.260	9.880	11.040	11.060
3F1P	78	17.520	17.840	15.860	17.073
3F1P	79	10.660	12.780	16.220	13.220
3F1P	80	16.020	17.960	11.140	15.040
**PDB-ID**	**Pose Number**	**HBS 1st traj**	**HBS 2nd traj**	**HBS 3rd traj**	**Average HBS**

2VXT	1	4.180	6.920	4.060	5.053
2VXT	2	5.120	3.680	7.740	5.513
2VXT	3	7.780	5.880	10.260	7.973
2VXT	4	14.720	6.940	4.280	8.647
2VXT	5	9.520	2.460	12.080	8.020
2VXT	6	8.280	8.580	2.520	6.460
2VXT	7	0.180	0.100	15.640	5.307
2VXT	8	8.580	11.920	10.640	10.380
2VXT	9	9.080	12.000	11.960	11.013
2VXT	10 (native)	18.420	18.300	18.200	18.307
2VXT	11	4.620	4.500	5.120	4.747
2VXT	12	5.080	3.860	6.320	5.087
2VXT	13	6.980	5.000	8.380	6.787
2VXT	14	5.740	4.680	8.760	6.393
**PDB-ID**	**Pose Number**	**HBS 1st traj**	**HBS 2nd traj**	**HBS 3rd traj**	**Average HBS**
3K75	15	15.560	19.300	10.480	15.113
3K75	16	8.300	17.060	11.680	12.347
3K75	17	16.860	18.980	15.360	17.067
3K75	18	8.520	18.860	21.740	16.373
3K75	19	13.340	12.720	11.960	12.673
3K75	20	28.000	21.220	20.960	23.393
3K75	21	7.880	12.080	13.660	11.207
3K75	22	3.860	7.620	6.680	6.053
3K75	23	6.980	9.180	7.460	7.873
3K75	24	19.520	10.200	20.960	16.893
3K75	25 (native)	9.000	6.840	7.700	7.847
3K75	26	9.660	17.320	15.100	14.027
3K75	27	2.600	6.300	9.100	6.000
3K75	28	16.580	11.860	10.220	12.887
3K75	29	19.360	10.580	7.560	12.500
3K75	30	7.720	12.640	5.640	8.667
3K75	31	16.120	12.860	13.660	14.213
3K75	32	6.300	8.560	7.160	7.340
3K75	33	7.380	7.540	8.320	7.747
3K75	34	11.020	1.520	10.220	7.587
**PDB-ID**	**Pose Number**	**HBS 1st traj**	**HBS 2nd traj**	**HBS 3rd traj**	**Average HBS**
4H03	35	14.860	19.000	10.000	14.620
4H03	36	14.920	12.340	19.860	15.707
4H03	37	12.780	13.240	18.940	14.987
4H03	38	15.800	11.320	9.900	12.340
4H03	39	12.200	8.460	17.060	12.573
4H03	40	16.180	15.600	15.740	15.840
4H03	41	21.640	25.580	17.120	21.447
4H03	42 (native)	8.660	15.780	13.980	12.807
4H03	43	7.080	6.780	3.780	5.880
4H03	44	18.340	18.960	15.340	17.547
4H03	45	8.720	13.460	10.400	10.860
4H03	46	16.760	6.200	13.040	12.000
4H03	47	19.780	20.680	15.040	18.500
4H03	48	14.560	17.400	15.920	15.960
4H03	49	17.580	8.360	13.440	13.127

4H03	50	23.660	8.440	9.060	13.720
4H03	51	15.160	6.340	10.420	10.640
4H03	52	10.640	12.060	9.800	10.833
4H03	53	20.260	11.940	15.520	15.907
4H03	54	12.360	4.840	11.980	9.727
**PDB-ID**	**Pose Number**	**HBS 1st traj**	**HBS 2nd traj**	**HBS 3rd traj**	**Average HBS**
4G6M	55 (native)	20.960	8.960	17.780	15.900
4G6M	56	2.280	4.580	5.440	4.100
4G6M	57	3.000	3.220	8.720	4.980
4G6M	58	3.580	4.440	6.520	4.847
4G6M	59	5.660	6.500	6.260	6.140
4G6M	60	7.100	2.040	7.040	5.393
4G6M	61	1.240	0.620	2.780	1.547
4G6M	62	5.660	5.760	5.260	5.560
4G6M	63	5.500	2.520	4.560	4.193
4G6M	64	6.620	6.360	7.060	6.680
4G6M	65	4.100	3.520	3.120	3.580
4G6M	66	1.180	3.240	1.720	2.047
4G6M	67	1.380	2.880	3.000	2.420
4G6M	68	4.740	3.180	3.060	3.660
4G6M	69	7.020	4.900	4.620	5.513
4G6M	70	4.200	0.560	2.880	2.547
4G6M	71	4.880	3.640	2.800	3.773
4G6M	72	6.380	2.620	3.540	4.180
4G6M	73	13.000	7.000	9.440	9.813
4G6M	74	5.160	8.540	5.960	6.553

Table S12: *average values of HBS/iRMSD calculated for each pose along three different SMD trajectories for the first 5ns (HBS/iRMSD 1st traj, HBS/iRMSD 2nd traj and HBS/iRMSD 3rd traj), and HBS/iRMSD values averaged on the three trajectories (Average HBS/iRMSD). The pose number highlighted in yellow represents the nearest native poses, having the lowest value of iRMSD*^{B} *(see Table S1). The highest values of HBS/iRMSD that identify the most stable pose are highlighted in yellow*

**PDB-ID**	**Pose Number**	**HBS/iRMSD 1st traj**	**HBS/iRMSD 2nd traj**	**HBS/iRMSD 3rd traj**	**Average HBS/ iRMSD**
1JTD	1 (native)	4.646	2.064	2.344	3.018
1JTD	2	1.793	1.821	1.446	1.687
1JTD	3	0.121	0.976	2.032	1.043
1JTD	4	0.329	0.200	0.240	0.257
1JTD	5	0.892	0.310	1.007	0.736
1JTD	6	0.414	2.034	0.619	1.022
1JTD	7	2.121	1.244	2.668	2.011
1JTD	8	0.397	1.930	1.312	1.213
1JTD	9	2.990	0.125	2.483	1.866

1JTD	10	1.665	2.667	1.547	1.959
1JTD	11	1.539	2.508	0.015	1.354
1JTD	12	0.395	0.554	0.286	0.412
1JTD	13	1.084	2.214	3.745	2.348
1JTD	14	3.078	0.466	4.162	2.569
1JTD	15	2.107	1.550	2.919	2.192
1JTD	16	2.030	1.284	2.327	1.880
1JTD	17	0.859	0.573	0.915	0.782
1JTD	18	0.149	2.151	0.788	1.029
1JTD	19	2.578	0.405	2.661	1.881
1JTD	20	3.426	2.142	2.428	2.665
**PDB-ID**	**Pose Number**	**HBS/iRMSD 1st traj**	**HBS/iRMSD 2nd traj**	**HBS/iRMSD 3rd traj**	**Average HBS/iRMSD**
2YVJ	21	3.160	2.933	5.300	3.798
2YVJ	22	4.176	6.423	1.569	4.056
2YVJ	23	3.710	3.391	0.585	2.562
2YVJ	24 (native)	5.672	2.255	3.775	3.901
2YVJ	25	5.305	6.300	5.927	5.844
2YVJ	26	2.087	5.702	4.748	4.179
2YVJ	27	0.968	0.697	1.832	1.165
2YVJ	28	1.026	1.350	0.913	1.096
2YVJ	29	5.900	5.679	3.451	5.010
2YVJ	30	3.310	2.318	1.295	2.308
2YVJ	31	3.769	3.600	1.476	2.948
2YVJ	32	2.164	0.593	3.625	2.127
2YVJ	33	1.053	1.217	1.417	1.229
2YVJ	34	2.138	3.786	0.293	2.072
2YVJ	35	2.056	5.382	2.767	3.401
2YVJ	36	3.214	4.444	3.837	3.831
2YVJ	37	2.670	4.853	1.740	3.087
2YVJ	38	1.422	0.998	0.965	1.128
2YVJ	39	1.649	2.838	3.304	2.597
2YVJ	40	2.714	2.943	3.668	3.109
**PDB-ID**	**Pose Number**	**HBS/iRMSD 1st traj**	**HBS/iRMSD 2nd traj**	**HBS/iRMSD 3rd traj**	**Average HBS/iRMSD**
3PC8	41 (native)	10.241	7.599	7.515	8.452
3PC8	42	2.992	2.073	2.266	2.444
3PC8	43	1.418	3.465	2.636	2.506
3PC8	44	1.577	1.047	1.055	1.226
3PC8	45	1.638	2.511	1.586	1.912
3PC8	46	0.506	0.480	0.688	0.558
3PC8	47	3.691	1.354	3.159	2.734
3PC8	48	6.409	3.260	5.729	5.133
3PC8	49	2.538	4.176	4.293	3.669
3PC8	50	5.523	3.352	5.350	4.742
3PC8	51	1.089	0.916	1.003	1.003
3PC8	52	3.620	6.392	2.094	4.035
3PC8	53	1.373	4.244	3.480	3.032
3PC8	54	0.940	0.848	2.936	1.575
3PC8	55	1.762	1.246	1.061	1.356
3PC8	56	3.287	3.701	3.263	3.417

3PC8	57	5.696	5.093	5.726	5.505
3PC8	58	3.164	1.895	2.098	2.385
3PC8	59	0.719	0.555	1.254	0.842
3PC8	60	2.773	6.435	1.868	3.692
**PDB-ID**	**Pose Number**	**HBS/iRMSD 1st traj**	**HBS/iRMSD 2nd traj**	**HBS/iRMSD 3rd traj**	**Average HBS/iRMSD**
3F1P	61	2.053	5.159	0.646	2.619
3F1P	62	4.649	1.387	2.228	2.755
3F1P	63	2.793	2.713	2.112	2.539
3F1P	64	2.127	2.841	3.757	2.909
3F1P	65	3.979	3.913	3.507	3.800
3F1P	66	2.926	3.225	2.961	3.037
3F1P	67	6.829	4.663	8.029	6.507
3F1P	68	2.119	2.391	1.499	2.003
3F1P	69	3.675	3.645	3.981	3.767
3F1P	70	3.414	3.143	2.307	2.955
3F1P	71	0.856	2.064	2.907	1.942
3F1P	72 (native)	8.046	6.946	10.085	8.359
3F1P	73	3.294	3.232	3.448	3.324
3F1P	74	3.100	4.147	3.798	3.682
3F1P	75	3.101	3.968	3.877	3.649
3F1P	76	2.038	0.690	1.179	1.302
3F1P	77	2.764	2.243	2.308	2.438
3F1P	78	6.393	6.085	5.337	5.938
3F1P	79	3.224	3.509	2.935	3.223
3F1P	80	3.428	4.890	2.076	3.465
**PDB-ID**	**Pose Number**	**HBS/iRMSD 1st traj**	**HBS/iRMSD 2nd traj**	**HBS/iRMSD 3rd traj**	**Average HBS/iRMSD**
2VXT	1	1.111	2.100	1.269	1.493
2VXT	2	1.014	0.524	2.272	1.270
2VXT	3	1.597	1.294	2.131	1.674
2VXT	4	5.980	2.449	0.945	3.124
2VXT	5	2.940	0.404	3.551	2.298
2VXT	6	2.620	2.446	0.510	1.859
2VXT	7	0.010	0.006	2.169	0.728
2VXT	8	2.822	3.862	3.694	3.459
2VXT	9	3.153	3.566	4.417	3.712
2VXT	10 (native)	5.649	5.552	5.413	5.538
2VXT	11	0.935	1.226	0.872	1.011
2VXT	12	1.508	0.806	1.835	1.383
2VXT	13	1.725	1.090	1.424	1.413
2VXT	14	0.791	0.754	1.549	1.031
**PDB-ID**	**Pose Number**	**HBS/iRMSD 1st traj**	**HBS/iRMSD 2nd traj**	**HBS/iRMSD 3rd traj**	**Average HBS/iRMSD**
3K75	15	1.902	3.596	3.079	2.859
3K75	16	3.646	0.365	3.496	2.502
3K75	17	5.710	4.314	8.585	6.203
3K75	18	1.300	3.005	3.395	2.567
3K75	19	2.124	1.721	2.509	2.118
3K75	20	4.077	4.045	2.826	3.649
3K75	21	1.864	4.395	2.846	3.035

3K75	22	4.519	7.969	5.683	6.057
3K75	23	5.535	3.499	3.789	4.275
3K75	24	0.587	1.828	2.643	1.686
3K75	25 (native)	6.939	7.244	3.809	5.997
3K75	26	3.997	2.824	1.803	2.875
3K75	27	2.856	6.061	5.181	4.699
3K75	28	2.343	3.447	2.332	2.707
3K75	29	2.609	1.718	1.297	1.875
3K75	30	1.042	3.258	2.583	2.294
3K75	31	2.028	2.490	1.871	2.130
3K75	32	3.955	4.400	3.798	4.051
3K75	33	11.903	5.952	7.983	8.613
3K75	34	0.731	0.565	1.404	0.900
**PDB-ID**	**Pose Number**	**HBS/iRMSD 1st traj**	**HBS/iRMSD 2nd traj**	**HBS/iRMSD 3rd traj**	**Average HBS/iRMSD**
4H03	35	3.940	4.849	3.266	4.018
4H03	36	4.446	2.368	5.256	4.023
4H03	37	3.253	3.406	4.340	3.666
4H03	38	4.563	2.920	2.463	3.315
4H03	39	2.939	1.714	3.880	2.844
4H03	40	5.855	4.598	5.824	5.426
4H03	41	3.773	8.614	5.594	5.994
4H03	42 (native)	2.188	5.060	4.068	3.772
4H03	43	1.566	1.019	0.720	1.102
4H03	44	3.961	4.871	3.443	4.091
4H03	45	2.115	2.255	2.537	2.302
4H03	46	5.595	0.904	4.425	3.642
4H03	47	5.382	7.031	4.193	5.535
4H03	48	3.453	6.072	5.015	4.847
4H03	49	5.403	2.698	5.426	4.509
4H03	50	6.647	2.142	2.096	3.628
4H03	51	3.803	1.247	3.122	2.724
4H03	52	2.052	1.616	1.653	1.774
4H03	53	6.289	3.131	4.935	4.785
4H03	54	4.481	1.048	4.404	3.311
**PDB-ID**	**Pose Number**	**HBS/iRMSD 1st traj**	**HBS/iRMSD 2nd traj**	**HBS/iRMSD 3rd traj**	**Average HBS/iRMSD**
4G6M	55 (native)	10.897	3.398	8.982	7.759
4G6M	56	1.288	1.863	1.336	1.495
4G6M	57	0.584	0.079	0.685	0.449
4G6M	58	1.801	0.632	1.419	1.284
4G6M	59	1.252	0.341	1.014	0.869
4G6M	60	1.077	1.180	2.193	1.483
4G6M	61	1.380	1.679	0.676	1.245
4G6M	62	0.098	0.470	0.402	0.323
4G6M	63	0.286	0.097	0.361	0.248
4G6M	64	0.491	0.511	1.651	0.884
4G6M	65	4.901	2.250	3.137	3.430
4G6M	66	1.136	0.863	1.047	1.016
4G6M	67	0.183	0.607	0.370	0.387
4G6M	68	2.073	1.474	2.418	1.989

4G6M	69	1.444	0.570	0.717	0.910
4G6M	70	0.796	0.960	0.821	0.859
4G6M	71	0.799	3.591	0.958	1.783
4G6M	72	0.491	0.930	0.896	0.772
4G6M	73	0.775	0.738	0.318	0.610
4G6M	74	1.754	1.343	0.995	1.364

References

(1) Dominguez. C.; Boelens. R.; Bonvin. A. M. J. J. HADDOCK: A Protein-Protein Docking Approach Based on Biochemical or Biophysical Information. *Journal of the American Chemical Society* **2003**. *125* (7). 1731–1737. https://doi.org/10.1021/ja026939x.

(2) van Zundert. G. C. P.; Rodrigues. J. P. G. L. M.; Trellet. M.; Schmitz. C.; Kastritis. P. L.; Karaca. E.; Melquiond. A. S. J.; van Dijk. M.; de Vries. S. J.; Bonvin. A. M. J. J. The HADDOCK2.2 Web Server: User-Friendly Integrative Modeling of Biomolecular Complexes. *Journal of Molecular Biology* **2016**. *428* (4). 720–725. https://doi.org/10.1016/j.jmb.2015.09.014.

(3) Lensink. M. F.; Méndez. R.; Wodak. S. J. Docking and Scoring Protein Complexes: CAPRI 3rd Edition. *Proteins: Structure. Function. and Bioinformatics* **2007**. *69* (4). 704–718. https://doi.org/10.1002/prot.21804.

(4) Janin. J.; Henrick. K.; Moult. J.; Eyck. L. ten; Sternberg. M. J. E.; Vajda. S.; Vakser. I.; Wodak. S. J. CAPRI: A Critical Assessment of PRedicted Interactions. *Proteins: Structure. Function. and Genetics* **2003**. *52* (1). 2–9. https://doi.org/10.1002/prot.10381.

(5) Vreven. T.; Moal. I. H.; Vangone. A.; Pierce. B. G.; Kastritis. P. L.; Torchala. M.; Chaleil. R.; Jiménez-García. B.; Bates. P. A.; Fernandez-Recio. J.; Bonvin. A. M. J. J.; Weng. Z. Updates to the Integrated Protein-Protein Interaction Benchmarks: Docking Benchmark Version 5 and Affinity Benchmark Version 2. *Journal of Molecular Biology* **2015**. *427* (19). 3031–3041. https://doi.org/10.1016/j.jmb.2015.07.016.

(6) Kyte. J.; Doolittle. R. F. A Simple Method for Displaying the Hydropathic Character of a Protein. *Journal of Molecular Biology* **1982**. *157* (1). 105–132. https://doi.org/10.1016/0022-2836(82)90515-0.

(7) Wimley. W. C.; White. S. H. Experimentally Determined Hydrophobicity Scale for Proteins at Membrane Interfaces. *Nature Structural Biology*. Nature Publishing Group October **1996**. 3. 842–848. https://doi.org/10.1038/nsb1096-842.

(8) Hessa. T.; Kim. H.; Bihlmaier. K.; Lundin. C.; Boekel. J.; Andersson. H.; Nilsson. I. M.; White. S. H.; von Heijne. G. Recognition of Transmembrane Helices by the Endoplasmic Reticulum Translocon. *Nature* **2005**. *433* (7024). 377–381. https://doi.org/10.1038/nature03216.

(9) Moon. C. P.; Fleming. K. G. Side-Chain Hydrophobicity Scale Derived from Transmembrane Protein Folding into Lipid Bilayers. *Proceedings of the National Academy of Sciences of the United States of America* **2011**. *108* (25). 10174–10177. https://doi.org/10.1073/pnas.1103979108.

(10) Zhao. G.; London. E. An Amino Acid “Transmembrane Tendency” Scale That Approaches the Theoretical Limit to Accuracy for Prediction of Transmembrane Helices: Relationship to Biological Hydrophobicity. *Protein Science* **2006**. *15* (8). 1987–2001. https://doi.org/10.1110/ps.062286306.
